# Apomictic Mountain Whitebeam (*Sorbus austriaca*, Rosaceae) Comprises Several Genetically and Morphologically Divergent Lineages

**DOI:** 10.3390/biology12030380

**Published:** 2023-02-27

**Authors:** Alma Hajrudinović-Bogunić, Božo Frajman, Peter Schönswetter, Sonja Siljak-Yakovlev, Faruk Bogunić

**Affiliations:** 1 Faculty of Forestry, University of Sarajevo, Zagrebačka 20, 71000 Sarajevo, Bosnia and Herzegovina; 2 Department of Botany, University of Innsbruck, Sternwartestrasse 15, 6020 Innsbruck, Austria; 3 Ecologie Systématique Evolution, CNRS, Université Paris-Sud, AgroParisTech, Université Paris-Saclay, 91400 Orsay, France

**Keywords:** apomixis, hybridisation, multiple origins, polyploidy, *Sorbus austriaca*

## Abstract

**Simple Summary:**

The genus *Sorbus* (whitebeams, rowans, and service trees) encompasses forest trees and shrubs characterised by exceptional diversity resulting from the interplay of polyploidisation, hybridization, and apomixis. The spatiotemporal processes driving *Sorbus* diversification remain poorly understood. This research aims to provide insights into the evolution and diversification patterns of mountain whitebeam (*S. austriaca*) covering most of its range in the mountains of Central and South-eastern Europe. Our molecular and morphometric data revealed pronounced cryptic diversity within the *S. austriaca* complex; it is composed of different lineages, that likely originated via multiple allopolyploidisations accompanied by apomixes, and these lineages exhibit different distribution patterns. Our results are particularly valuable from a biodiversity conservation perspective due to the continuing generation of novel diversity in sympatric populations of the parental taxa. Such derived diversity requires process-oriented conservation plans and measures.

**Abstract:**

The interplay of polyploidisation, hybridization, and apomixis contributed to the exceptional diversity of *Sorbus* (Rosaceae), giving rise to a mosaic of genetic and morphological entities. The *Sorbus austriaca* species complex from the mountains of Central and South-eastern Europe represents an allopolyploid apomictic system of populations that originated following hybridisation between *S. aria* and *S. aucuparia*. However, the mode and frequency of such allopolyploidisations and the relationships among different, morphologically more or less similar populations that have often been described as different taxa remain largely unexplored. We used amplified fragment length polymorphism (AFLP) fingerprinting, plastid DNA sequencing, and analyses of nuclear microsatellites, along with multivariate morphometrics and ploidy data, to disentangle the relationships among populations within this intricate complex. Our results revealed a mosaic of genetic lineages—many of which have not been taxonomically recognised—that originated via multiple allopolyploidisations. The clonal structure within and among populations was then maintained via apomixis. Our results thus support previous findings that hybridisation, polyploidization, and apomixis are the main drivers of *Sorbus* diversification in Europe.

## 1. Introduction

The evolutionary importance of polyploidisation in flowering plants as a key mechanism shaping plant diversity is widely acknowledged [1,2,3,4]. Whole-genome multiplication via auto- or allopolyploidy can reshuffle genome structure, alter gene expression, induce phenotypic and physiological changes, and provide adaptive potential to polyploid plants [5,6]. Polyploidisation is sometimes connected to the breakdown of self-incompatibility systems and allows a transition from outcrossing to asexual (uniparental) reproduction [7]. Apomixis, which is asexual reproduction involving seed formation, has proven to be an effective strategy in the evolution of certain plant groups, as it preserves and maintains hybrid and heterozygous genetic lineages, as well as cytotypes with unbalanced chromosomes, enabling their long-term persistence and dispersal [8,9]. Apomictic polyploids produce clonal offspring genetically identical to the mother plant; most, however, retain residual sexuality (facultative apomixis [10]).

In most apomicts, the combination of factors such as clonality, residual sexuality, and multiple origins affects both geographic distribution and genotypic diversity within populations [11,12]. Apomicts show a better adaptive capacity and greater colonisation ability by often occupying extreme ecological niches or disturbed habitats. Furthermore, they may have larger distribution ranges than their sexual relatives, a phenomenon known as ‘geographical parthenogenesis’ [9,13,14,15,16]. While some apomictic species have broad ranges, there are numerous examples of apomict species with narrow ranges, such as *Rubus* L. [17], *Sorbus* L. [18,19], or *Taraxacum* F.H. Wigg [20]. The distribution ranges of apomictic species, however, largely depend on the applied species concept, either treating single or a few apomictic populations as distinct species [19,21,22,23,24,25,26] or lumping them into more broadly distributed morphospecies [27,28]. In addition, many hybridogenous apomictic complexes originated via polytopic allopolyploidisation events, e.g., in *Crataegus* L. [29], *Potentilla* L. [30], *Rubus* [31], or *Taraxacum* [12], but also in the intricate genus *Sorbus* [32,33].

*Sorbus* (whitebeams, rowans, and service trees, amongst others) encompasses trees and shrubs characterized by exceptional genetic and morphological diversity resulting from the interplay of polyploidisation, hybridisation, and apomixis [34,35,36,37]. It includes about 190 species inhabiting the Northern Hemisphere [19]. Diversification of European *Sorbus* has been primarily driven by hybridisation of four widely distributed diploids, namely *S. aria* (L.) Crantz, *S. aucuparia* L., *S. chamaemespilus* (L.) Crantz, and *S. torminalis* (L.) Crantz, and backcrossing of hybrids with their parental species, which led to the formation of many allopolyploid apomictic lineages [18,34,38]. Most hybrid derivatives are tri- and tetraploids that reproduce by apomixis and are restricted to geographical areas of varying sizes; many of them have been described as distinct species [18,26,39,40]. In addition, a small portion of the polyploids in *Sorbus* originated via autopolyploidisation [33,36].

Nevertheless, most of the *Sorbus* polyploids are facultative apomicts, and only a few are obligate apomicts [37,41]. The most common scenario for the formation of novel diversity involves mixed-ploidy communities consisting of species and cytotypes with different mating systems and their interaction through interploid gamete exchange, often resulting in polyploid offspring [41]. The recurrent interaction of hybridisation and polyploidisation generates hybrid swarms of intermediate morphology that are stabilised through apomixis and represent an inexhaustible source of *Sorbus* diversity. This, however, often results in complicated and unresolved taxonomies [42] vs. [43]. Treating morphologically recognizable and apomictically reproducing triploid and tetraploid entities of monophyletic origin as distinct species has been a species concept adopted by most recent authors [18]. These species often have restricted distributions, frequently confined to single localities in which they likely originated, thus representing single clones or related clone mates [42]. Such species concepts resulted in the description of numerous apomictic taxa in different parts of Europe [18,23,24,26,44,45].

*Sorbus* subgen. *Soraria* Májovský and Bernátova is one of the five hybridogenous subgenera, which include diploid and polyploid species that originated from hybridisation between *Sorbus* subgen. *Sorbus* and *Sorbus* subgen. *Aria* Pers. One of its members, *Sorbus austriaca* (Beck) Hedl. (Austrian whitebeam or mountain whitebeam [46]), is a tetraploid obligate pseudogamous apomict species [37,41]. It was described from the valley Rettenbachgraben, close to the village of Prein an der Rax (Niederösterreich, Austria [47]), but based on the morphological similarity of other populations considered to be distributed in the Eastern Alps, the Balkan Peninsula, and the Carpathians [19,48], it has a relatively large range compared with most other species of subgen. *Soraria*. However, the circumscription of this species and its differentiation from other species of *Sorbus* subgen. *Soraria* is unclear, and its precise distribution is thus unknown [19,49].

Initially, Austrian whitebeam was treated as a variety or subspecies of *S. mougeotii* Soyer-Willemeet and Godron [*S. mougeotii* var. *austriaca* (G. Beck) C.K. Schneider; *S. mougeotii* subsp. *austriaca* (G. Beck) Hedl.] or even as a subspecies of *S. aria* [*S. aria* subsp. *austriaca* (G. Beck) Bornm.]. After its recognition as a species, it was split into four subspecies: *S. austriaca* subsp. *austriaca*, *S. austriaca* subsp. *croatica* Kárpáti, *S. austriaca* subsp. *hazslinszkyana* (Soó) Kárpáti, and *S. austriaca* subsp. *serpentini* Kárpáti [46]. However, the most recent taxonomic treatments do not support the recognition of subspecific taxa [19,26,42]. Consequently, Kurto et al. [19] treated subsp. *hazslinszkyana*, distributed in Slovakia and northern Hungary, as a distinct species, *S. hazslinszkyana* (Soó) Boros. Furthermore, they included *S. austriaca* subsp. *serpentine*, distributed in Eastern Austria, in the *S. aria* species complex and excluded subsp. *croatica* distributed in Croatia, as an unresolved name. The *S. austriaca* species complex thus represents an allopolyploid apomictic system of populations that originated following hybridisation between *S. aria* and *S. aucuparia* in the mountains of Central and South-Eastern Europe, but with unclear relationships.

Besides *S. austriaca*, the only morphologically similar tetraploid species with a large distribution in Europe is *S. mougeotii*, ranging from Spain to western Austria, where it is adjacent to the western distribution margin of *S. austriaca* [19,46]. In addition, other morphologically similar tetraploid species with more narrow distributions have been described from Central Europe, e.g., *S. lippertiana* N. Mey. and Meierott, endemic to Germany and Austria, with main distribution in the north-eastern Limestone Alps, but ranging also to the south-eastern Limestone Alps [40], *S. pekarovae* Májovský and Bernátová, stenoendemic to the Žilina region in Slovakia [50], and *S. pauca* M. Lepší and P. Lepší, stenoendemic to the Doksy region in Czech Republic [25]. Furthermore, similar species described outside of Central Europe include *S. anglica* Hedl. (endemic to Great Britain and Ireland, mainly south-western England and Wales [18]), *S. cuneifolia* T.C.G. Rich (stenoendemic to Great Britain, distributed in Denbighshire county, Wales [22]), and *S. subsimilis* Hedl. (endemic to South Norway [51]). However, since many of the narrow endemic species were described solely on morphological and karyological grounds, nothing is known about their phylogenetic relationships, exact circumscription, and distribution patterns.

The aim of this study is to provide insights into relationships and diversification patterns within *S. austriaca* as currently circumscribed [19] as well as its relationships to other species belonging to the complex. We sampled 21 localities, covering most of the distribution of *S. austriaca* (*sensu* Kurtto et al. [19]), but also including four other described microspecies (one locality per microspecies) from Central and Western Europe, as well as three localities of *S. mougeotii* for comparison. More specifically, we aimed to (1) reconstruct the phylogenetic relationships among the sampled populations of *Sorbus* subgen and *Soraria* and in relation to their parents, *S. aria* and *S. aucuparia*, using amplified fragment length polymorphism (AFLP) fingerprinting, nuclear microsatellites, and plastid DNA sequencing. Furthermore, (2) we inferred the studied populations’ ploidy and reproductive mode using flow cytometry; (3) we tested self-compatibility as a component of the reproductive system; and (4) we explored the geographic distribution and morphological variation of the apomictic lineages. We tested the hypothesis that different populations within the group represent independent evolutionary entities and thus represent hitherto cryptic diversity. We do not aim to provide a revised taxonomy of this group and name all evolutionary entities—which we consider premature—but rather discuss the taxonomic implications of our results.

## 2. Materials and Methods

### 2.1. Plant Material

**Figure 1:**
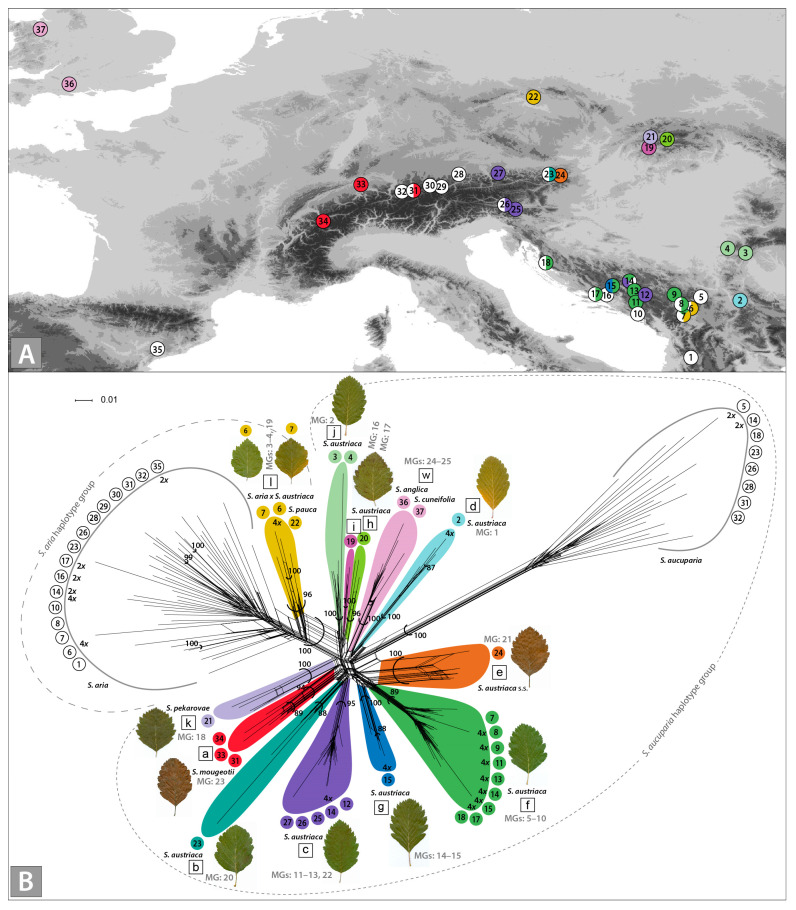
Geographic origin (**A**) and amplified fragment length polymorphism (AFLP) variation (**B**) of analysed *Sorbus* accessions. Sampled localities are numbered; details are in Table S1. AFLP clusters within *Sorbus* subgen. *Soraria* are colour-coded and labelled with the letters *a–l*; in (**A**) their presence in each sampled locality is indicated (the parental taxa *S. aria* and *S. aucuparia* are in white). The NeighborNet diagram is supplemented with bootstrap values > 85% derived from a Neighbour-joining analysis (Figure S1); multilocus genotypes (MG’s) derived from nuclear microsatellites; and ploidy data obtained by flow cytometry; dashed lines denote the plastid haplotype affiliation according to the plastid *trnT*–*trnF* phylogenetic analysis. Note that only a subset of individuals was sequenced for plastid DNA variation.

Leaf material from 37 localities was collected and silica-dried for molecular analyses and herbarized for morphological analyses (Figure 1A, Table S1); localities 22, 27, 36, and 37 were sampled only for molecular analyses. The taxa were identified using *Flora Europaea* [46], Euro + Med Plantbase [52], and national floras [18,53,54,55,56]. Voucher specimens are kept at the National Museum of Bosnia and Herzegovina (SARA).

### 2.2. Amplified Fragment Length Polymorphism (AFLP)

One to 13 individuals per locality, totaling 172 individuals from 37 sampled localities, were included in the AFLP analysis (Table S1). A modified CTAB-procedure [57] was used for extraction of total genomic DNA from c. 20 mg of silica-dried leaf material. The AFLP protocol was followed [58], with the modifications described in [39]. We used the following primer combinations for the selective PCR (fluorescent dye in brackets): *Eco*RI (6-FAM)-ACA/*Mse*I-CAC, *Eco*RI (VIC)-AAG/*Mse*I-CTG, and *Eco*RI (NED)-ACC/*Mse*I-CAG (*Mse*I- and *Eco*RI-primers: Sigma-Aldrich). Reproducibility was tested using fifteen replicated samples. Electropherograms were analysed with Peak Scanner 1.0 (Applied Biosystems, Waltham, MA, USA) with default peak detection parameters. The minimum fluorescent threshold was set at 50 relative fluorescence units (RFU). RawGeno 2.0 [59], a package for R [60], was used for automated data scoring with the following settings: 75–500 bp scoring range, 50 RFU minimum intensity, bin width 1.0–1.5. Fragments with reproducibility lower than 80% based on sample-replicate comparisons were excluded. A neighbour-joining analysis based on Nei-Li genetic distances [61] was conducted and bootstrapped (2000 pseudo-replicates) with TREECON 1.3 b [62]. A NeighborNet was produced from a matrix of uncorrected P distances using SplitsTree 4.12 [63]. A principal coordinate analysis (PCoA) based on Jaccard distances was conducted using PAST 2.15 [64].

### 2.3. Analysis of Nuclear Microsatellites

The amplification of six nuclear microsatellite-specific loci (CH01F02, MSS5, MSS13, MSS16, D11, and H10) was successfully performed for 171 individuals from 37 sampled localities (Table S1), following Robertson et al. [33,34]. An ABI PRISM 310 Genetic Analyzer (Applied Biosystems) was used for electrophoretic separation of the PCR products. Alleles were sized relative to the internal size standard TAMRA 500 (Applied Biosystems). Electropherograms were analysed using GeneMapper (Applied Biosystems). To study the genetic diversity, we determined the multilocus genotype (MG) for each individual on the basis of microsatellite alleles for each of the six loci using the software GenoType 1.2 [65]. Assignment of individuals to a particular clone was completed using the algorithm of Meirmans and Van Tienderen [65] according to the calculation of a genetic distance matrix and a threshold value (set to 2 after testing different thresholds as recommended) under the stepwise mutation model option. Clonal diversity was presented as the sum of the total number of multilocus genotypes (*Ng*), effective number of genotypes (*Eff*) and genotypic diversity (*Div*), calculated with GenoDive 1.2 [65]. Relationships among multilocus genotypes were visualised by principal coordinate analysis (PCoA) based on Jaccard distances using PAST 3.17 [64]. The maximum number of alleles per locus was used to infer the ploidy level.

### 2.4. Plastid trnT–trnF Sequencing and Phylogenetic Analyses

The plastid *trnT–trnF* region was sequenced for 39 individuals from 31 localities (GenBank number in Table S1), following the procedure described by Hajrudinović et al. [39] and using the primers TabA, TabC and TabF [66]; in addition, 15 sequences were included from Hajrudinović et al. [39]. Sequences were edited and aligned with Geneious Pro 5.5.9 [67]. We coded indels as binary characters using simple gap coding [68] with SeqState 1.25 [69].

Maximum parsimony (MP) and MP bootstrap (MPB) analyses of the concatenated plastid sequences (including gap codes) were performed using PAUP 4.0b10 [70]. The most parsimonious trees were searched for heuristically with 100 replicates of random sequence addition, TBR swapping, and MulTrees on. All characters were equally weighted and unsorted. The data set was bootstrapped using full heuristics with 1000 replicates, TBR branch swapping, MulTrees option off, and a random addition sequence with five replicates. Bayesian analyses of the same dataset were performed using MrBayes 3.2.1 [71], applying the HKY85 substitution model proposed by the Akaike information criterion implemented in MrAIC.pl 1.4 [72]. The alignment was partitioned into nucleotide and indel data sets, and the latter was treated as morphological data according to the model of Lewis [73]. Values for all parameters, such as the shape of the gamma distribution, were estimated during the analyses. The settings for the Metropolis-coupled Markov chain Monte Carlo process included four runs with four chains each (three heated ones using the default heating scheme), running simultaneously for 10,000,000 generations each, sampling trees every 1000th generation using default priors. The posterior probabilities (PP) of the phylogeny and its branches were determined from the combined set of trees, discarding the first 1001 trees of each run as burn-in.

### 2.5. Genome Size Estimation

Genome size estimation followed the protocol of Hajrudinović et al. [41], using flow cytometry. Briefly, fresh leaves of 45 individuals from 12 populations (Table S1) were co-chopped with a razor blade with fresh leaves of the internal standard *Medicago truncatula* Gaertn. cv. R108-1 (0.98 pg [74]) in 600 mL of cold Gif nuclear buffer. The suspension was filtered through a 50 μm nylon mesh (CellTrics, Partec), and RNAse (Roche) was added to 25 U mL^−1^. The nuclei were stained with propidium iodide (Sigma-Aldrich) with a final concentration of 50 mg mL^−1^ and incubated on ice for ca. 15 min prior to analysis. The fluorescence of ~3000 nuclei was recorded for each sample using a Partec CyFlow SL3 (Partec, Münster, Germany) 532 nm laser cytometer or CyFlow Ploidy Analyser (Sysmex Europe SE) 532 nm laser. The 2C DNA values were obtained, and DNA ploidy levels [75] were inferred by comparison with the 2C DNA values of individuals of known chromosome counts, i.e., 2*n* = 2*x* = 34 for diploids and 2*n* = 4*x* = 68 for tetraploids [76].

For the purpose of reproduction mode identification, we conducted flow cytometric seed screening (FCSS) on 40 seeds from 10 locations (see Results), following Hajrudinović et al. [41]. Seeds were collected from previously cytotyped mother individuals (Table S1). Furthermore, to test for self-compatibility, the inflorescences of three *S. austriaca* trees from locality 14 were covered with pollination bags before anthesis. Seven seeds from pollination bags were collected and analysed. Only well-formed seeds, cleaned, shortly dried at room temperature, and kept in paper bags at 4 °C prior to analysis were used. Each seed was analysed separately. Endosperm ploidy was calculated using the inferred monoploid genome size of the embryo. Following Hajrudinović et al. [41], DNA ploidies of embryo and endosperm were compared with distinguish between sexual and apomictic origin of each seed.

### 2.6. Morphometric Analyses

Morphometric measurements were performed on 96 individuals from 24 localities (Table S1) of simple-leaved populations belonging to *S. aria* × *S. austriaca*, *S. austriaca*, *S. mougeotii* and *S. pekarovae* (all from the subgenus *Soraria*). Leaves of *S. anglica*, *S. cuneifolia*, and *S. pauca*, distributed outside the range of *S. austriaca*, were not available for measurements. The measurements included leaf characters that were previously shown to be informative [18,23,24,27,77]. The following 18 quantitative leaf characters were measured: lamina length (LLEAV), petiole length (LPET), length of the first, second, and third tooth (1SEINL, 2SEINL, 3SEINL), length of the first, second, and third nerve (1NERV, 2NERV, 3NERV), angle between the first, second, and third nerve compared with the primary nerve (1NANG, 2NANG, 3NANG), lamina width (WLEAV), distance of the leaf base to the line of maximal leaf width (MXWLEAV), leaf width 1 cm beneath the leaf apex (1ALEAV), leaf width 1 cm above the leaf base (1BLEAV), number of secondary nerve pairs (NNER), ratio of lamina length and lamina width (LLEAV/WLEAV) and ratio of lamina width and distance of the leaf base to the line of maximal leaf width (WLEAV/MXLEAV). The measurements were completed by hand, using millimeter paper and a digital calliper. The arithmetic means of three to five measurements per leaf character for each individual (from different mid-leaves of short sterile shots) were used for statistical analyses. Multivariate principal component analysis (PCA), canonical discriminant analysis (CDA), and classificatory discriminant analysis (DA) were performed for two data matrices. The first matrix encompassed data of all samples (96 individuals), while *S. pekarovae*, *S. mougeotii* and three putative back-crossed individuals *S. aria* × *S. austriaca* (AFLP group *l* in Figure 1B) were excluded from the second matrix (84 individuals of the *S. austriaca* lineages, see Results). Two canonical discriminant analyses (CDA 1 and CDA 2) followed by two classificatory discriminant analyses (DA 1 and DA 2) were performed for the two data matrices [78]. Prior to the analyses, the data matrix was standardised due to the different measurement units used. PCA based on the correlation matrix characters of the first matrix was aimed at displaying a general pattern of variation and relationships among individuals/populations/taxa. CDA based on Mahalanobis’ distances was used to analyse the morphology of *a priori* defined groups using the 12 AFLP groups with simple leaves marked as *a–l* in Figure 1B. The obtained results were validated using DA. The validation criterion in the identification of morphological groups was >70% of *a posteriori* correctly classified cases into the *a priori* defined groups in CDA [79]. Classificatory DA was performed using a leave-one-out cross-validation (jackknifing) procedure. Both analyses were performed using PAST 3.14 [64]. Furthermore, basic descriptive statistical parameters were calculated for the analysed taxa/genetic groups: the arithmetic mean (µ), standard deviation (SD), value range (Min–Max) and coefficient of variation (CV%).

## 3. Results

### 3.1. AFLP Fingerprinting

We obtained 435 high-quality and reproducible AFLP fragments from 172 individuals. The initial average error rate was 4.1%. The neighbour-joining analysis inferred 15 clusters with high bootstrap support (BS > 85%; Figure S1) that were also divergent in the NeighborNet (Figure 1B). The two most divergent clusters contained the parental species, *S. aucuparia* and *S. aria*. Whereas the former cluster was genetically more uniform, separated by a long split from all other samples, the latter cluster was more diverse. All other 13 clusters that were positioned intermediate between the parental taxa corresponded to *Sorbus* subgen. *Soraria*. Populations treated as *S. austriaca* were included in nine clusters, which included single populations scattered in the Northeastern Limestone Alps (Austria; clusters *b* and *e*, the latter including population 24 sampled ten kilometres away from the *locus classicus* of *S. austriaca*), the central Balkan Peninsula (Bosnia and Herzegovina, *g*; Serbia, *d*), and the Western Carpathians (Slovakia, *h*, *i*). Cluster *j* included two populations from the Southern Carpathians (Romania), cluster *c* included five populations from the Northern and Southern Limestone Alps (Austria, Slovenia) and the central Balkan Peninsula (Bosnia and Herzegovina), and cluster *f* included nine populations from the western Balkan Peninsula (Dinaric Mountains; Bosnia and Herzegovina, Croatia, Kosovo, and Serbia). Three populations of *S. mougeotii* from the Western Alps (Switzerland) and the Northern Limestone Alps (Austria) were included in cluster *a*. Likewise, the single population of *S. pekarovae* from the Western Carpathians (Slovakia) formed its own cluster *k*, whereas the single populations of *S. anglica* and *S. cuneifolia* from England and Wales, respectively, were included in cluster *w*. Finally, two populations from the central Balkan Peninsula (Kosovo and Serbia) and the single individual of *S. pauca* from the eastern Sudetes (Czech Republic) were genetically closest to *S. aria* and were included in cluster *l*; we assume these individuals are hybrids between *S. aria* and *S. austriaca*.

### 3.2. Nuclear Microsatellites

**Figure 2:**
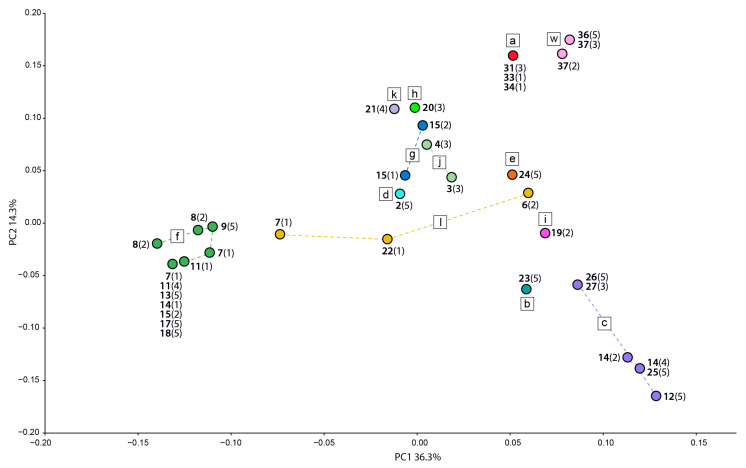
Principal coordinate analysis of Jaccard distances among the 25 multilocus genotypes found in 110 accessions of *Sorbus* subgen. *Soraria* accessions based on nuclear microsatellite data. Locality numbers correspond to Figure S1A and Table 1 and colours and letters to the AFLP clusters in Figure 1B. Multilocus genotypes belonging to the same AFLP cluster are connected with dashed lines. The numbers in parentheses denote the number of clones per locality.

A total of 25 clonal multilocus genotypes (MGs) were found within 110 accessions of *Sorbus* subgen. *Soraria* (Figure 2, Table 1). Twenty-four out of 28 localities contained at least one MG shared by a different number of individuals within a locality, whereas localities 22 (Czech Republic), 33, and 34 (Switzerland) contained only one sampled individual, and locality 7 (Kosovo) contained three unique genotypes (Table S2). Nineteen populations with >1 sampled individuals were completely clonal (*Ng* = 1; Table S2). The effective number of genotypes (*Eff*) was higher than 1 in six populations due to the presence of unique genotypes conferring higher values of genotypic diversity (*Div*) in those populations. Nineteen MGs were limited to single populations, and MG 2 was present in geographically close populations (Table 1). However, several MGs occurred at multiple localities (MGs 2, 5, 12, 22, 23, and 24; Table 1). The most widespread clonal genotype (MG 5, Table 1) occurred at seven localities in the Dinaric Mountains (localities 7, 11, 13, 14, 15, 17, and 18; Table 1).

The relationships among MGs (Figure 2) were generally consistent with the AFLP data (Figure 1B). The two most divergent *Soraria* MG clusters along the first PCoA axis thus corresponded to AFLP clusters *c* and *f*, including populations from the Balkan Peninsula and the Eastern Alps. The populations of most other AFLP clusters were scattered between these two main MG clusters; clusters *a* and *w* were most divergent from cluster *c* along the second PCoA axis. The AFLP cluster *l* including putative hybrids *S. austriaca* × *S. aria* was most diverse. Individuals from locality 14 (Bosnia and Herzegovina) had the most heterogeneous genotypes. Individuals were included in the two most divergent clusters, in accordance with the AFLP results, where these individuals were included in clusters *c* and *f*. The populations from other areas were more uniform, however, without a clear geographic pattern. There were also several monoclonal populations, namely in locations 19, 20, and 21 from the Western Carpathians, 23 and 24 from the Eastern Alps, and 2 from the Central Balkan Peninsula. On the other hand, the populations from locations 3 and 4 from the Southern Carpathians and 31, 33, and 34 from the Alps all shared a single clone. In addition, the allele composition of the parental species, *S. aucuparia* and *S. aria*, is given in Table S3.

### 3.3. Plastid trnT–trnF Phylogenetic Relationships

**Figure 3:**
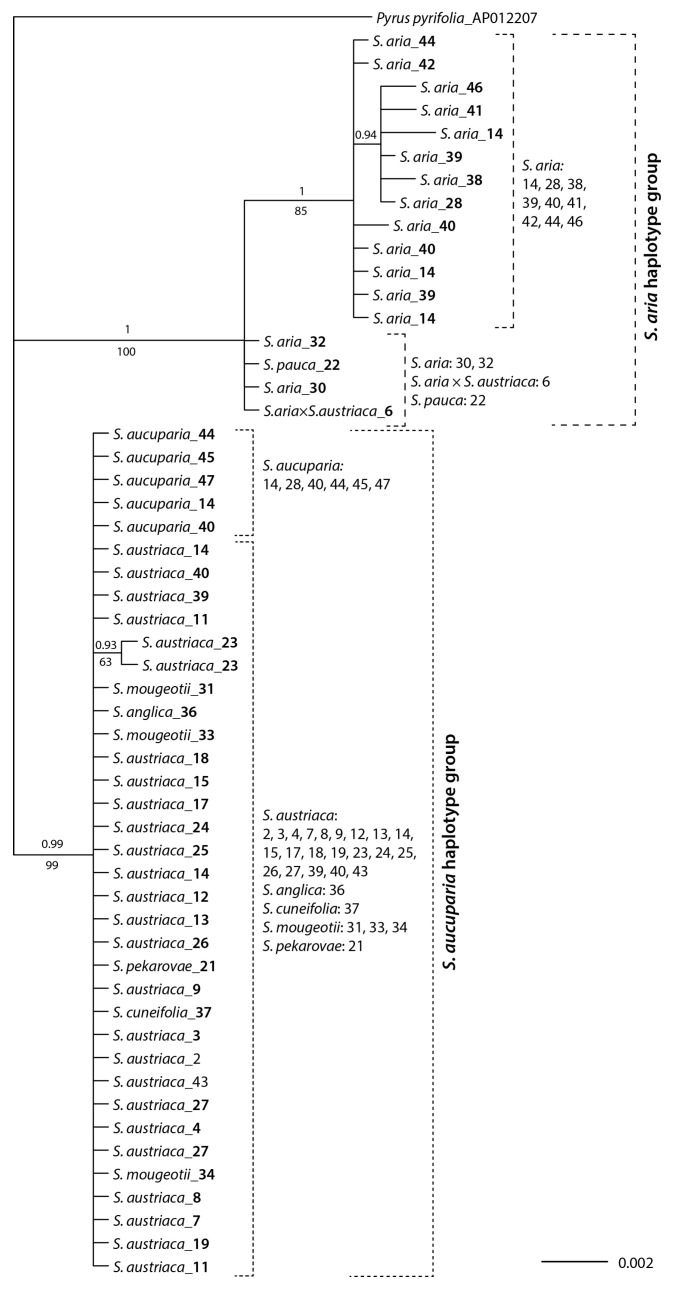
Bayesian consensus phylogram inferred from phylogenetic analyses of plastid *trnT–trnF* sequences. Numbers above branches are posterior probabilities > 0.90 and those below branches maximum parsimony bootstrap values > 50%. Locality numbers correspond to Table S1.

The *trnT-trnF* alignment of the concatenated *trnT*-*trnL* intergenic spacer and the *trnL-trnF* partial sequence was 1943 bp long. The shortest sequences (1766 bp) were those of *S. anglica*, *S. aucuparia*, *S. cuneifolia*, *S. mougeotii*, *S. pekarovae*, and almost all of *S. austriaca*; they were all identical. Two individuals of *S. austriaca* from locality 23 were 8 bp longer. The sequences of *S. aria*, including also one of *S. pauca* and one of *S. aria* × *S. austriaca* varied much more in length, ranging from 1817 bp (*S. aria* from locality 41) over 1838 bp (*S. aria* 30, 32; *S. aria* × *S. austriaca* 6; *S. pauca* 22), 1840 bp (*S. aria* 14, 28, 39, 40, 42, 44, 46) to 1850 bp (*S. aria* 38), 1860 bp (*S. aria* 40) and 1864 bp (*S. aria* 14). Eight substitutions, of which two were outapomorphic, contributed to variability within the *S. aria* lineage. Twenty-three characters were parsimony-informative, and Bayesian and parsimony analyses of the *trnT–trnF* sequences resulted in congruent phylogenies (Figure 3) that reflected the variation outlined above.

Two main clades were resolved: one (posterior probability, pp, 1; parsimony bootstrap, BS, 100%), named *S. aria* haplotype group, included two populations of *S. aria* (localities 30 and 32), one of *S. aria* × *S. austriaca* (locality 6), and one of *S. pauca* (locality 22) in a basal polytomy, and a clade (PP 1, BS 85%) including all other populations of *S. aria* (*S. aria* haplotype group; localities 14, 28, 38–42, 44, 46). The second main clade (PP 0.99, BS 99%), named *S. aucuparia* haplotype group, included all populations of *S. aucuparia* (localities 14, 44, 45, 47) and *S. austriaca* (localities 2–4, 7–9, 12–15, 17–19, 23–26), the single populations of *S. anglica* (locality 36), *S. cuneifolia* (locality 37), and *S. pekarovae* (locality 21), as well as the three populations of *S. mougeotii* (localities 31, 33, 34), with unresolved relationships.

### 3.4. Genome Size, Ploidy Level and Reproduction Mode

**Figure 4:**
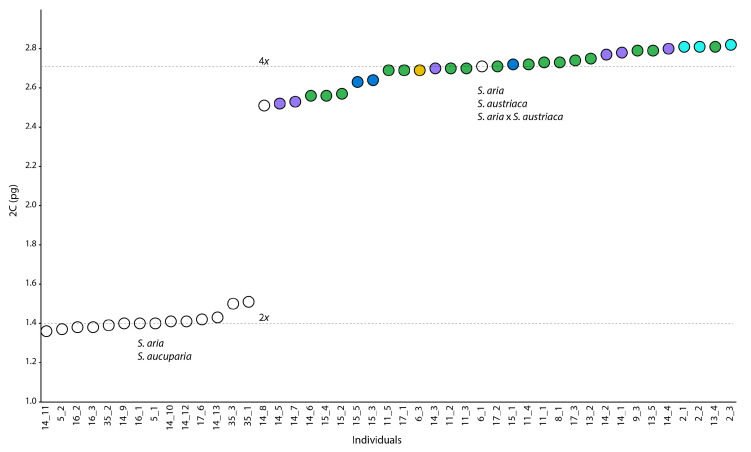
Scatterplot of absolute genome size values (2C pg) for analysed *Sorbus* accessions. Colours correspond to the AFLP clusters (Figure 1B) and locality numbers followed by individual numbers to Figure 1A and Table S1.

Flow cytometry of 45 individuals resulted in holoploid absolute genome sizes (GS, 2C value) corresponding to two ploidy levels, namely diploid and tetraploid (Figure 4).

The GS was 1.36–1.43 pg in diploid *S. aucuparia*, 1.38–1.51 pg in diploid *S. aria*, 2.69 pg in tetraploid *S. aria × S. austriaca*, 2.52–2.82 pg in tetraploid *S. austriaca*, and 2.51–2.71 pg in tetraploid *S. aria* (Table 2).

Flow cytometric seed screening of 40 seeds resulted in three different embryo:endosperm profiles, namely 2*x*:3*x*, 4*x*:10*x* and 4*x*:12*x* (Table 2). Diploid *S. aria* and *S. aucuparia* mother trees yielded seeds with 2*x* embryo and 3*x* endosperm, which represents a regular sexual profile. On the other hand, all seeds of analysed tetraploid *S. austriaca*, *S. aria* and *S. aria* × *S. austriaca* mother trees were of apomictic origin with 4*x* embryos and 12*x* endosperms, or, in one seed, 10*x* endosperm (Table 2). Moreover, several inflorescences of the three *S. austriaca* individuals from locality 14 that were covered with pollination bags to check for self-compatibility, yielded fruits. All seeds developed in pollination bags were of apomictic origin with 4*x* embryos and 12*x* endosperms (Table 2).

### 3.5. Morphology

Principal Component Analysis generated four significant principal components (two are displayed, Table S4). They accounted for 83.1% of the total variance (PC1 = 40.2%, PC2 = 19.6%, PC3 = 14.1%, and PC4 = 9.1%), with moderate correlation to the majority of corresponding morphological traits (Table S4). The PCA ordination diagram (Figure S2) showed a pattern in which most AFLP clusters overlapped among the four clusters (*c*, *d*, *f*, and *k*). The CDA 1 diagram of all samples (Figure S3A) showed that the overlapping AFLP clusters *c* (Dinaric Mountains, Southern Limestone Alps, Northern Limestone Alps), *h*, *i*, and *j* (all Carpathians) were morphologically divergent from the overlapping clusters *e* (Northern Limestone Alps), *f* and *g* (both Dinaric Mountains), and *l* (Dinaric Mountains, Bohemia), whereas the partly overlapping Alpine clusters *a* (Western Alps) and *b* (Northern Limestone Alps) were intermediate along DF1 (explaining 41.5% of variation). Along DF2 (explaining 20.6% of the variation), the populations from AFLP clusters *d* (Carpathians-Balkan Mountains) and *k* (Carpathians) were clearly divergent, whereas all other AFLP clusters were intermediate. Finally, along DF3 (11.3%; Figure S3B), some AFLP clusters that were overlapping along DF1 and DF2 were divergent, namely clusters *a*, *b*, *e*, and *j*. The characters that contributed mostly to the discrimination along DF1 were LLEAV/WLEAV, NNERV, 1NERV and 2NERV, those along DF2 were WLEAV, LLEAV/WLEAV, WLEAV, and those along DF3 1NANG, 2NANG. The classification matrix with the Jackknifed procedure for the CDA 1 dataset resulted in 82% of correctly classified individuals (Table S5).

**Figure 5:**
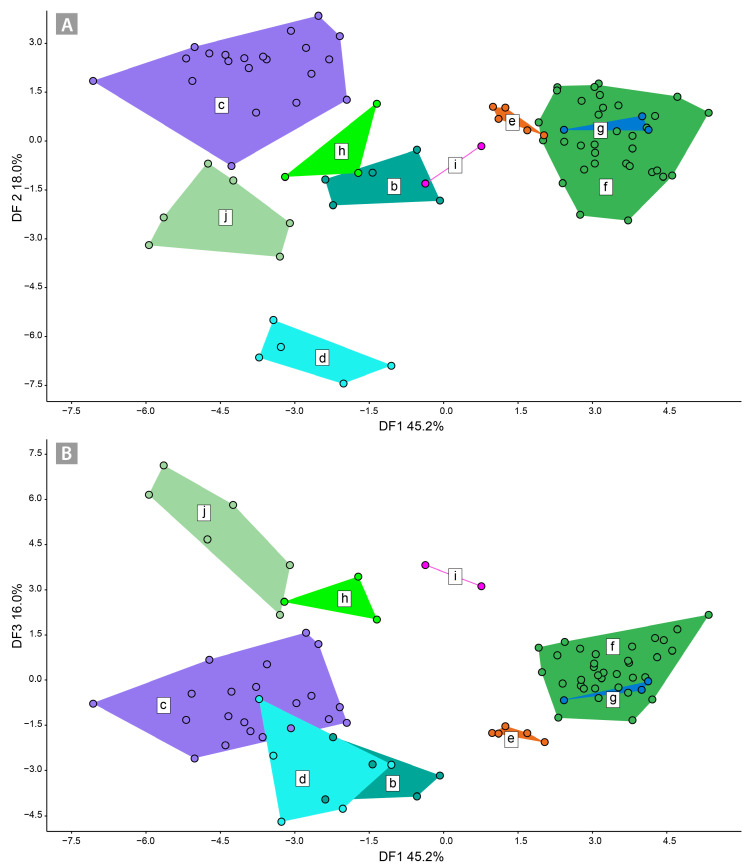
Canonical discriminant analysis ((**A**) DF1 vs. DF2; (**B**) DF1 vs. DF3) of nine predefined *Sorbus austriaca* groups (corresponding to the AFLP clusters shown in Figure 1) based on 18 morphological leaf characters.

The CDA 2 resulted in clearer morphological discrimination among the AFLP clusters of the *S. austriaca* lineages (Figure 5). Along with DF1 (45.2%) and DF2 (18.0%; Figure 5A), AFLP clusters *c*, *d*, and *j* were clearly divergent. On the other hand, *f*, *e*, and *g*, as well as *b*, *h*, and *i*, overlapped. Along DF3 (16.0%; Figure 5B) clusters *e*, *h*, *j* and *j* were divergent, whereas *c*, *d*, and *f*, as well as *f* and *g*, overlapped. The characters that contributed most to the discrimination along DF1 were LLEAV/WLEAV, 1NERV, NNER, and 2NERV; those along DF2 were WLEAV, LLEAV/WLEAV, NNERV, and 3NER; and those along DF3 were WLEAV/MXLEAV. The classification matrix with the Jackknifed procedure for the CDA 2 dataset resulted in 88% of correctly classified individuals (Table S6). Basic descriptive statistical parameters of measured leaf characters are given in Table S7.

## 4. Discussion

Our integrative approach combining AFLP fingerprinting, plastid DNA sequences, nuclear microsatellites, ploidy-level estimation, and morphometric analyses inferred intricate patterns of diversification within the *S. austriaca* complex. The genetic data revealed a clear divergence among (groups of) populations included in different allotetraploid apomictic lineages that originated in various parts of South-Eastern and Central Europe. Our results thus support previous findings that hybridisation, polyploidization, and apomixis are the main drivers of *Sorbus* diversification, at least in Europe [33,34,36,82].

### 4.1. Multiple Origins of S. austriaca Lineages

Our AFLP and nuclear microsatellite data suggest independent origins of the different *S. austriaca* lineages in different parts of the Alps, Dinaric Mountains, and the Carpathians, likely as a consequence of polytopic hybridisation between the parental diploid species *S. aria* and *S. aucuparia* (Figure 1B and Figure 2, Table 1). Hybridisation was followed by polyploidisation, as all individuals of *S. austriaca* for which we established the ploidy, were tetraploid (Figure 1B and Figure 4, Table 1 and Table 2). This pattern, along with the restriction of many lineages to single (lineages *b*, *d*, *e*, *g*, *h*, *i*, and *k*) or a few (lineages *a*, *j*, and *w*) localities, some of them in areas that were strongly glaciated during the Last Glacial Maximum (LGM) [83,84], may imply their recent origin. On the other hand, the geographically widespread lineages *c* and *f* included multiple populations and shared related multiclonal MGs differing in one or two alleles per locus (Figure 1A,B; Table 1). Cluster *f* includes populations solely from the Dinaric Mountains, which were much less affected by the Pleistocene glaciations than the Alps [85,86]. Cluster *c* also includes populations from this area and the southern margins of the Eastern Alps, which suggests that they might have originated earlier and thus had more time to disperse [87]. Their disjunct distributions could be a result of multiple dispersals and establishments in isolated localities, but also of a previously more continuous range disrupted by the climatic changes during the Pleistocene.

An important factor in the range expansion of apomicts is self-compatibility, a reproductive trait of the mating system of *S. austriaca* as determined in population 14 (Table 2). Self-compatibility most likely facilitated the range expansion of certain clonal genotypes, more specifically those from clusters *c* and *f* (Figure 1B). Due to self-compatibility, apomictic clones can establish populations via single individuals that use their own pollen for the required endosperm fertilisation to produce functional seeds [7]. In this way, apomictic genotypes promote range expansions to remote areas, where they can function as pioneer colonisers of new habitats [15]. The fleshy fruits of *Sorbus*, such as those of other *Malinae* (e.g., *Crataegus*), are adapted to dispersal by vertebrates, mainly birds, often over relatively long distances [16,88].

A certain level of genetic differentiation among the populations in the Dinaric cluster *f* (Figure 1) suggests their persistence in disjoint localities over a longer period rather than their recent dispersal. Such a differentiation is surprising, as it is not expected in an obligate apomictic such as *S. austriaca* [37,41]. Different mechanisms can facilitate genetic divergence even among obligate apomictic populations, namely residual sexuality [89], accumulation of mutations and chromosome rearrangements [12,90], recombination during restitutional meiosis [91], transposon activity [92], and heritable epigenetic variation [93]. On the other hand, in the Dinaric-Alpine cluster *c*, the level of diversification appears to be lower, suggesting more recent divergence of some populations. This is consistent with the extensive glaciation of the Alps during the LGM [94], rendering postglacial colonisation of the Alpine populations from the South likely. Nuclear microsatellite analysis confirmed that diploids from South-eastern Europe (Albania and Montenegro) and Central Europe (Slovenia) were involved in the formation of the Central European tetraploid populations of *S. aria* [87], which further supports the hypothesis of postglacial colonisation of the Alps from the South. In the same line, the Balkan Peninsula served as an important Pleistocene refugium and a source for post-glacial colonisation of Central Europe for other trees, e.g., alder buckthorn (*Frangula alnus* L. [95]), European beech (*Fagus sylvatica* L. [96]), and hornbeam (*Carpinus betulus* L. [97]).

The co-occurrence of the widespread genetic lineage *f* and the stenoendemic lineages *c* and *g* at the same localities in the central Dinaric Mountains (14 and 15, Figure 1) is an additional line of evidence for recurrent allopolyploidisations in sympatric populations of parental diploid sexuals. Weak reproductive barriers between *S. aucuparia* and *S. aria* facilitate gene flow and continuously generate novel hybrid derivatives, which are often polyploid [32]. Along the same line, the co-occurrence of AFLP group *f* in *S. austriaca* and *S. aria* at locality 7 in the south-eastern Dinaric Mountains could have led to hybridisation and, consequently, the origin of a novel genetic entity (i.e., cluster *l*).

### 4.2. Mostly S. aucuparia, but also S. aria, Served as Maternal Parents of Hybridogenous Lineages

As in most angiosperms [98], plastomes are maternally inherited in *Sorbus* [99]. Our plastid DNA phylogenies clearly show that hybridogenous populations of *S. anglica*, *S. austriaca*, *S. cuneifolia*, *S. mougeotii*, and *S. pekarovae* all had *S. aucuparia* as their maternal parent (Figure 1A and Figure 3), which is the most common pattern in the subgenus *Soraria* [32,34,39,81,100]. *Sorbus aria* acts as a pollen donor not only in hybrids with *S. aucuparia* but also with *S. torminalis* and *S. chamaemespilus* [32,34,39,100], albeit with some exceptions [88].

Also in our dataset, AFLP cluster *l* that was in the NeighborNet closest to *S. aria* (Figure 1B), shared its haplotype with *S. aria* (Figure 3). Due to their intermediate position between *S. aria* and the *S. austriaca* complex in the NeighborNet, we suggest that these populations represent backcrosses of the *S. austriaca* complex with *S. aria* as the maternal parent (*S. aria* × *S. austriaca*). Alternatively, direct hybridisation of *S. aria* with *S. aucuparia* as a pollen donor could be plausible, but we consider this less probable because such a scenario would result in an intermediate leaf morphology (semipinnate leaves [33]). To our best knowledge, *S. aucuparia* served as the maternal parent to all allopolyploid derivatives originating from hybridisation with *S. aucuparia*.

The individuals of cluster *l* in our study, including *S. pauca*, all shared a *S. aria* haplotype and were tetraploids with simple leaves (Figure 1B, Table 2). Whereas localities 6 and 7 are separated by only 27 km, and the clustering of their individuals is thus not surprising, the close relationship of the single analysed individual of *S. pauca* from a locality more than 970 km away, is unexpected. *Sorbus pauca* was recently described as a hybridogenous tetraploid apomictic endemic of the eastern Sudetes that most probably arose from two hybridisation events [25], but this hypothesis as well as the formal description of this new species were exclusively based on morphology and cytometry. It is likely that multiple recent hybridisations between genetically similar parents at different localities led to highly similar hybrid genotypes. Alternatively, cluster *l* has a single origin and is more common in intermediate areas, for instance the Carpathians.

The origin of *Sorbus* tetraploids may follow two different pathways. One involves an initial cross of a diploid sexual and a tetraploid apomict, leading to apomictic triploid offspring. Subsequently, unreduced triploid eggs are fertilised by reduced pollen of the parental diploid to produce new tetraploids [33,37,41]. Alternatively, crossing the reduced megagametophyte (*n* = 2*x*) of a sexual tetraploid with the reduced pollen (*n* = 2*x*) of an apomictic tetraploid can produce the same tetraploid offspring. In the case of *S. austriaca*, the first scenario would include fertilisation of a reduced diploid *S. aucuparia* megagametophyte (*n = x*) with reduced pollen of tetraploid *S. aria* (*n = 2x*) to produce triploid offspring; tetraploid *S. aria* is namely widespread in the Balkan Peninsula [39,41]. In the next step, the unreduced megagametophyte of such a triploid is fertilised by reduced pollen of *S. aucuparia* (*n = x*) to produce a tetraploid. Facultative sexuality of apomictic allopolyploids could allow backcrossing with parental species [101]. The second scenario would include fertilisation of a reduced allotetraploid megagametophyte (such as *S. bosniaca* with the same parental combination [39]) with reduced pollen of tetraploid *S. aria*. The problem with the latter scenario is that there is no evidence for sexuality in *S. bosniaca*. The third scenario presumes a direct cross via fertilisation of an unreduced *S. aucuparia* megagametophyte (*n* = 2*x*) with reduced pollen of tetraploid *S. aria* (*n = 2x*).

Therefore, different scenarios for the formation of *Sorbus* polyploids are plausible, at least in the Dinaric Mountains, where different cytotypes and taxa often coexist [39,41]. Despite recent methodological progress, reconstructing the origin of allopolyploids is still challenging due to their recurrent formation, recombination among homeologous chromosomes, different epigenetic expression, genome restructuring, or extinction of parental lineage [5,6,102,103]. It is particularly difficult to trace subgenomes of the *S. aria* group within allopolyploid complexes due to the enormous genetic and cytotypic variability of the members of this group [82].

### 4.3. Taxonomic Considerations

Taxonomic assessments are a serious challenge in apomictic groups, such as *Amelanchier* Medik. [104], *Antennaria* Gaertn. [105], *Crataegus* [106], *Taraxacum* [12], *Hieracium* L. [107], *Ranunculus* L. [28], *Rubus* [17], and *Sorbus*. The taxonomic concept adopted by most European taxonomists in the past two decades in *Sorbus* has been the morphospecies concept, which implies a unique morphology coupled with distributional data [107]. Numerous apomictic species (microspecies) in Europe have been described based on unique morphology combined with cytometric or karyological and sometimes genotypic data [21,23,24,26,40,44,45,108,109].

In *Sorbus*, particularly ploidy data are considered important because in this genus, polyploidy confers apomictic reproduction, which is the key criterion in addition to morphology [18]. Apomictic reproduction *per se* does not necessarily imply uniform and distinctive morphology but may result in poorly differentiated individuals/populations arising from the same or related combinations of parental taxa/cytotypes, as shown for *S. aria* from Central Bavaria [82]. Our results show that genetically similar populations also tend to cluster together morphologically, regardless of their geographic origin (clusters *c* and *f*, Figure 1B, Figures S2 and S3).

Our study reveals cryptic diversity within the *S. austriaca* complex. High congruence of molecular and morphological data demonstrates that the sampled populations of *S. austriaca* in fact form multiple evolutionary entities. Interestingly, both the level of genetic (Figure 1B) and morphological (Figures S2 and S3) divergence among described species of subgenus *Soraria*, namely *S. anglica* and *S. cuneifolia* (cluster *w*), *S. mougeotii* (*a*), and *S. pekarovae* (*k*), is similar to the divergence among different lineages of *S. austriaca* (clusters *b*–*g*). The *S. austriaca* clusters are differentiated from *S. pekarovae* and *S. mougeotii* based on leaf morphology (Figure S3), although some morphological overlap between *S. mougeotii* and *S. austriaca* (cluster *b*, population 23) is evident.

The strongest morphological differentiation is seen among *S. austriaca* lineages from the Balkan Peninsula (clusters *f* and *d*) and the Balkan-Alpine cluster *c* (Figure 5A). The Balkan populations have genetic affinities with both the Northern and Southern Limestone Alps (cluster *c*, Figure 1A and Figure 5), which is also reflected in similar morphology. On the other hand, the pronounced genetic and morphological divergence of the Carpathian lineages of *S. austriaca* (the Southern Carpathian cluster *j* and the Western Carpathian clusters *i* and *h*) is in line with their geographical isolation relative to lineages from the Alps and the Dinaric Mountains. Their distinctiveness may be explained by a potentially different parental combination, i.e., a non-sampled paternal lineage of genetically variable *S. aria* [45,82].

Allopolyploidisation likely occurs polytopically, leading to the evolution of independent populations [81], and natural selection and chromosome rearrangements can then result in the formation of similar morphological forms [110]. On the other hand, epigenetic mechanisms coupled with polyploidy can produce different phenotypes regardless of the similarity of the genomic compositions of allopolyploids [111]. In our case, the morphological integrity of the *S. austriaca* lineages and their distinctiveness across the sampled area are likely maintained via apomixis and reproductive isolation. Even if some of the genetic clusters could be associated with existing species (e.g., cluster *c* could pertain to the recently described *S. lippertiana* [40]), the others likely represent undescribed cryptic microspecies. We here refrain from recognising the uncovered cryptic diversity as distinct taxonomic entities; additional studies, including a denser sampling and more detailed morphological analyses, are needed to taxonomically resolve this intricate complex.

## 5. Conclusions

Our molecular data revealed pronounced cryptic diversity within the *S. austriaca* complex; it is actually composed of different lineages, which likely originated at different time horizons and exhibit different distribution patterns. These data highlight the importance of genetic analyses on the one hand and including samples from a broader geographic area when taking taxonomic decisions and describing new species in *Sorbus* on the other hand. Apart from that, our results are particularly valuable from a biodiversity conservation perspective, because in the genus *Sorbus*, the interaction of hybridisation, polyploidy, and apomixis represents a powerful mechanism generating novel diversity in sympatric populations of the parental taxa. Traditional conservation efforts, based on clearly defined boundaries of taxonomic entities in order to specify appropriate action plans and measures, have limitations in preserving the diversity of taxonomic complex groups [112], such as the genus *Sorbus*, whose dynamic evolution requires a different approach. Therefore, a new conservation concept based on evolutionary processes was proposed (Process-Based Species Action Plan [113]). The goal of this concept is the conservation of processes that generate diversity, i.e., the preservation or increase of the number of individuals in a potential interaction. Studies such as the present one are timely, as they are setting the stage for such process-oriented biodiversity conservation.

## Figures and Tables

**Table 1:** Multilocus genotypes (MGs) and allele composition of the studied 28 populations of *Sorbus* subgen *Soraria*.

MG	N	AFLP Cluster	N Per Locality	*Loci*						*Xo*	*Xe*
				MSS13	MSS5	CH01F02	D11	MSS16	H10		
**1**	5	d	**2**(5)	189, 193, 195	124, 126, 130	186, 200	143, 151	154, 158, 162	86	3	**4*x* ^a^**
**2**	6	j	**3**(3), **4**(3)	193, 197	110, 124, 126	186, 190, 196, 200	137, 151	154, 158, 160, 162	92	**4**	-
**3**	2	l	**6**(2)	191, 193, 200	124, 134	194, 196	149, 151	154, 158, 160	82, 88, 92	3	**4*x* ^a^**
**4**	1	l	7(1)	193, 195, 201	122, 124, 126, 130	190, 196, 198, 200	151, 157	156, 158	-	**4**	**-**
**5**	23	f	**7**(1), **11**(4), **13**(5), **14**(1), **15**(2), **17**(5), **18**(5)	191, 195, 201	124, 126, 150	186, 190, 196, 200	143, 151	156, 158	104, 106	**4**	**4*x* ^a^**
**6**	1	f	**7**(1)	191, 195, 201	124, 126, 150	186, 190, 196, 200	143, 151	154, 158	104, 106	**4**	-
**7**	2	f	**8**(2)	193, 197, 201	124, 126	186, 196	143, 151	156, 158	104, 106	3	**4*x* ^a^**
**8**	2	f	**8**(2)	191, 197, 201	124, 126	186, 190, 196	143, 151	156, 158	106	3	**4*x* ^a^**
**9**	5	f	**9**(5)	191, 197, 201	124, 126	186, 190, 196	143, 151	154, 158, 166	104, 106	3	**4*x* ^a^**
**10**	1	f	**11**(1)	191, 195, 201	124, 126, 150	186, 190, 196, 200	143, 151	156, 158	102, 104	**4**	**4*x* ^a^**
**11**	5	c	**12**(5)	191, 193, 195	110, 112, 124	188, 196, 202	147, 153	154, 158, 162	92	3	-
**12**	9	c	**14**(4), **25**(5)	191, 193, 195	110, 112, 124	188, 196, 202	147, 151	154, 158, 162	92	3	**4*x* ^a^**
**13**	2	c	**14**(2)	191, 193, 195	110, 112, 124	188, 196, 202	147, 151	154, 158, 162	80, 90	3	**4*x* ^a^**
**14**	2	g	**15**(2)	191, 193	112, 120, 126	186, 190, 196, 200	151	154, 158, 164	98, 100	**4**	**4*x* ^a^**
**15**	1	g	**15**(1)	191, 193	112, 120, 126	186, 190, 196, 200	151	154, 158, 164	102	**4**	**4*x* ^a^**
**16**	2	i	**19**(2)	191, 193, 195	112, 120, 126	188, 190, 196, 200	147, 149, 151	154, 158, 160, 162	92	**4**	-
**17**	3	h	**20**(3)	-	110, 112, 126	186, 190, 196, 200	149, 151	154, 160	96	**4**	**4*x* ^d^**
**18**	4	k	**21**(4)	193, 201	124, 126, 132	186, 190, 196	131, 151	154, 162	90	3	**4*x* ^d^**
**19**	1	l	**22**(1)	191, 193, 201	112, 124, 126	190, 196, 200	149, 151	156, 158, 160, 162	92	**4**	**4*x* ^c^**
**20**	5	b	**23**(5)	191, 193, 195	110, 112, 124	186, 196, 208	131, 151	154, 158, 162	102, 104	3	-
**21**	5	e	**24**(5)	191, 193, 195	124	190, 196	141, 147, 151	154, 158, 160	98, 100	3	-
**22**	8	c	**26**(5), **27**(3)	191, 193, 195	110, 112, 124	186, 196, 200	147, 151	154, 158, 162	92	3	-
**23**	5	a	**31**(3), **33**(1), **34**(1)	193, 197, 201	124, 132	186, 196	147, 151	154, 160, 162	100, 102	3	-
**24**	8	w	**36**(5), **37**(3)	191, 193, 195	118, 128, 134	186, 196, 200	147, 149, 151	154, 162	98, 100	3	**4*x* ^b^**
**25**	2	w	**37**(2)	191, 193, 195	118, 128, 134	186, 196, 200	147, 149, 151	154, 162	96, 98	3	**4*x* ^b^**
	**110**										

N—Number of individuals belonging to each multilocus genotype; N per locality—Number of individuals (in brackets) bearing a particular multilocus genotype per each locality (in bold), numbered as in Figure 1; *Xo*—Maximum number of alleles per locus; *Xe*—Expected ploidy level–obtained by flow cytometry for at least one individual per population from each MG group **^a^** or from published results for the same sampled locality [80] **^b^**, [25] **^c^**, [81] **^d^**.

**Table 2:** Flow cytometric results for nuclei from leaves and seeds of *Sorbus* taxa accompanied with the deduced origin of seeds/reproduction mode.

Locality No.	Affiliation in AFLP NNet	Taxon	*N*	Genome Size (2C pg)	Genome Size (1C*x* pg)	DNA Ploidy Level (2*n*)	*N* Seeds	Embryo (2C pg)	Endosperm (2C pg)	Embryo: Endosperm Ploidy	Seed Origin
2	*d*	*S. austriaca*	3	2.81–2.82	0.70–0.71	4*x*	-				
5	*S. aucuparia*	*S. aucuparia*	2	1.37–1.40	0.69–0.70	2*x*	2	1.36–1.41	2.07–2.14	2*x*:3*x*	Sexual
6	*l*	*S. aria × S. austriaca*	1	2.69	0.67	4*x*	1	2.67	8.13	4*x*:12*x*	Apomictic
	*S. aria*	*S. aria*	1	2.71	0.68	4*x*	1	2.77	8.15	4*x*:12*x*	Apomictic
8	*f*	*S. austriaca*	1	2.73	0.68	4*x*	1	2.73	8.08	4*x*:12*x*	Apomictic
9	*f*	*S. austriaca*	1	2.79	0.70	4*x*	1	2.73	8.10	4*x*:12*x*	Apomictic
11	*f*	*S. austriaca*	5	2.69–2.73	0.67–0.68	4*x*	1	2.72	8.32	4*x*:12*x*	Apomictic
13	*f*	*S. austriaca*	3	2.75–2.81	0.69–0.70	4*x*	2	2.73–2.74	8.09–8.23	4*x*:12*x*	Apomictic
14	*f*	*S. austriaca*	1	2.56	0.64	4*x*	1	2.81	8.46	4*x*:12*x*	Apomictic
	*c*	*S. austriaca*	6	2.52–2.80	0.63–0.70	4*x*	19 (7 *)	2.69–2.80	7.99–8.65	4*x*:12*x*	Apomictic
							1	2.65	6.70	4*x*:10*x*	Apomictic
	*S. aria*	*S. aria*	2	1.40–1.41	0.70–0.71	2*x*	2	1.41–1.43	2.12–2.15	2*x*:3*x*	Sexual
			1	2.51	0.63	4*x*	1	2.74	8.34	4*x*:12*x*	Apomictic
	*S. aucuparia*	*S. aucuparia*	3	1.36–1.43	0.68–0.72	2*x*	3	1.36–1.41	2.03–2.13	2*x*:3*x*	Sexual
15	*f*, *g*	*S. austriaca*	5	2.56–2.72	0.64–0.68	4*x*	1	2.67	8.12	4*x*:12*x*	Apomictic
16	*S. aria*	*S. aria*	3	1.38–1.40	0.69–0.70	2*x*	2	1.37–1.43	2.06–2.13	2*x*:3*x*	Sexual
17	*f*	*S. austriaca*	3	2.69–2.74	0.67–0.69	4*x*	1	2.66	8.11	4*x*:12*x*	Apomictic
	*S. aria*	*S. aria*	1	1.42	0.71	2*x*	-				
35	*S. aria*	*S. aria*	3	1.39–1.51	0.70–0.76	2*x*	-				
			45				40 (7 *)				

* Asterisks mark the number of seeds from inflorescences isolated in pollination bagss.

## Data Availability

The data presented in this study are available within article and Supplementary Materials.

## Supplementary Materials

The following supporting information can be downloaded at: https://www.mdpi.com/article/10.3390/biology12030380/s1, Figure S1, Neighbor-Joining analysis of AFLP data for *Sorbus* subgen. *Soraria*, *S.* subgen. *Aria* and *S.* subgen. *Sorbus*. Localities’ numbers and groups’ colours follow the other Tables and Figures; Figure S2, PCA ordination of 18 morphological traits for all *Soraria* accessions. Colours and letters correspond to AFLP clusters in Figure 1; Figure S3, Canonical discriminant analysis (A: DF1 vs. DF2; B: DF1 vs. DF3) of 12 predefined groups (corresponding to AFLP clusters) based on individual plants and 18 morphological characters. Colours and letters correspond to AFLP clusters in Figure 1; Table S1, Geographic origin and number of individuals of *Sorbus* populations included in the analyses of AFLP, nuclear microsatellite, plastid DNA sequencing, flow cytometric and morphometric data, respectively; Table S2, Indices of population clonal diversity based on nuclear microsatellite data for the studied populations of *Sorbus* subgen. *Soraria*; Table S3, Alelle composition in six nuclear microsatellite loci for the presumed parental taxa of *Sorbus* subgen. *Soraria*; Table S4, Results of principal component analysis (PCA, see Figure S2) and canonical discriminant analysis (CDA, see Figure S3) of *Soraria* individuals based on the morphological characters of leaves; Table S5, Classification matrix with Jackknife procedure (82% of cases correctly classified) for the dataset of analysed *Sorbus* individuals based on morphometric leaf measurements (groups are defined according to AFLP clusters, Figure 1B); Table S6, Classification matrix with Jackknife procedure (88% of cases correctly classified) for the dataset of analysed *Sorbus austriaca* individuals based on morphometric leaf measurements (groups are defined according to AFLP clusters, Figure 1B); Table S7, Descriptive statistics of leaf morphometrics for *Sorbus* subgen. *Soraria* individuals presented for AFLP groups.
