# Supplementary material for: Apomictic Mountain Whitebeam (Sorbus austriaca, Rosaceae) Comprises Several Genetically and Morphologically Divergent Lineages
Source: Biology (Basel). 2023 Feb 27;12(3):380. doi: 10.3390/biology12030380 (PMC10045669; doi:10.3390/biology12030380)
Supplement: Supplementary file 1 [file biology-12-00380-s001.zip › Hajrudinovic et al_Biology_2023_SUPPLEMENTARY.pdf]

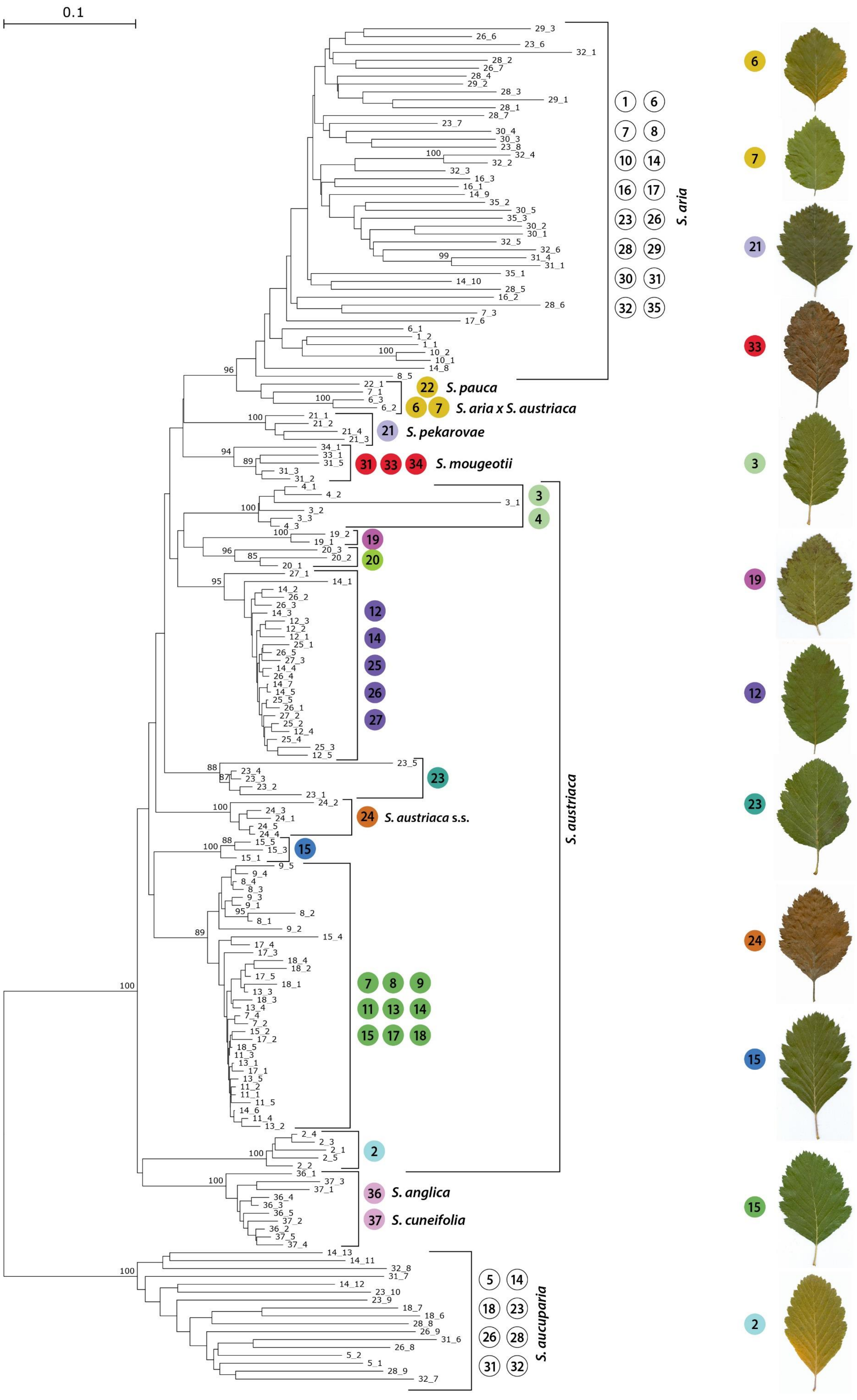

Figure S1. Neighbor-Joining analysis of AFLP data for *Sorbus* subgen. *Soraria*, *S.* subgen. *Aria* and *S.* subgen. *Sorbus*. Localities' numbers and groups' colours follow the other Tables and Figures.

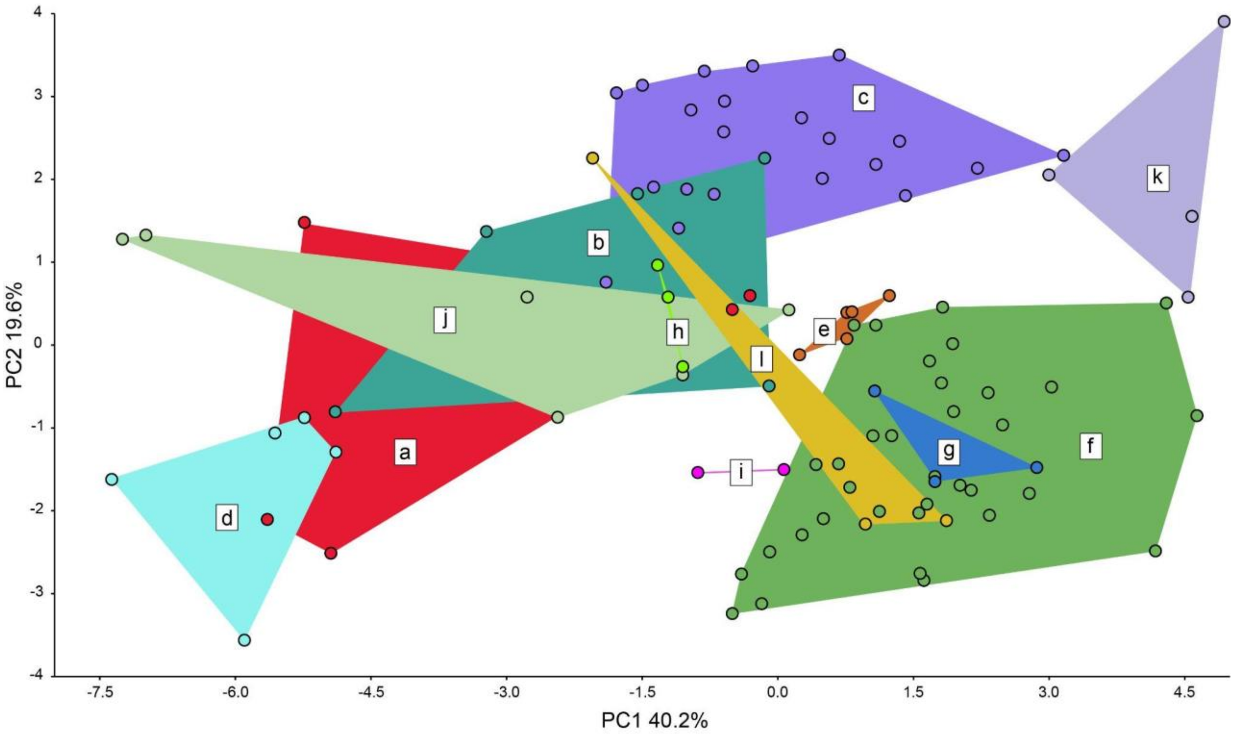

Figure S2. PCA ordination of 18 morphological traits for all *Soraria* accessions.Colours and letters correspond to AFLP clusters in Figure 1.

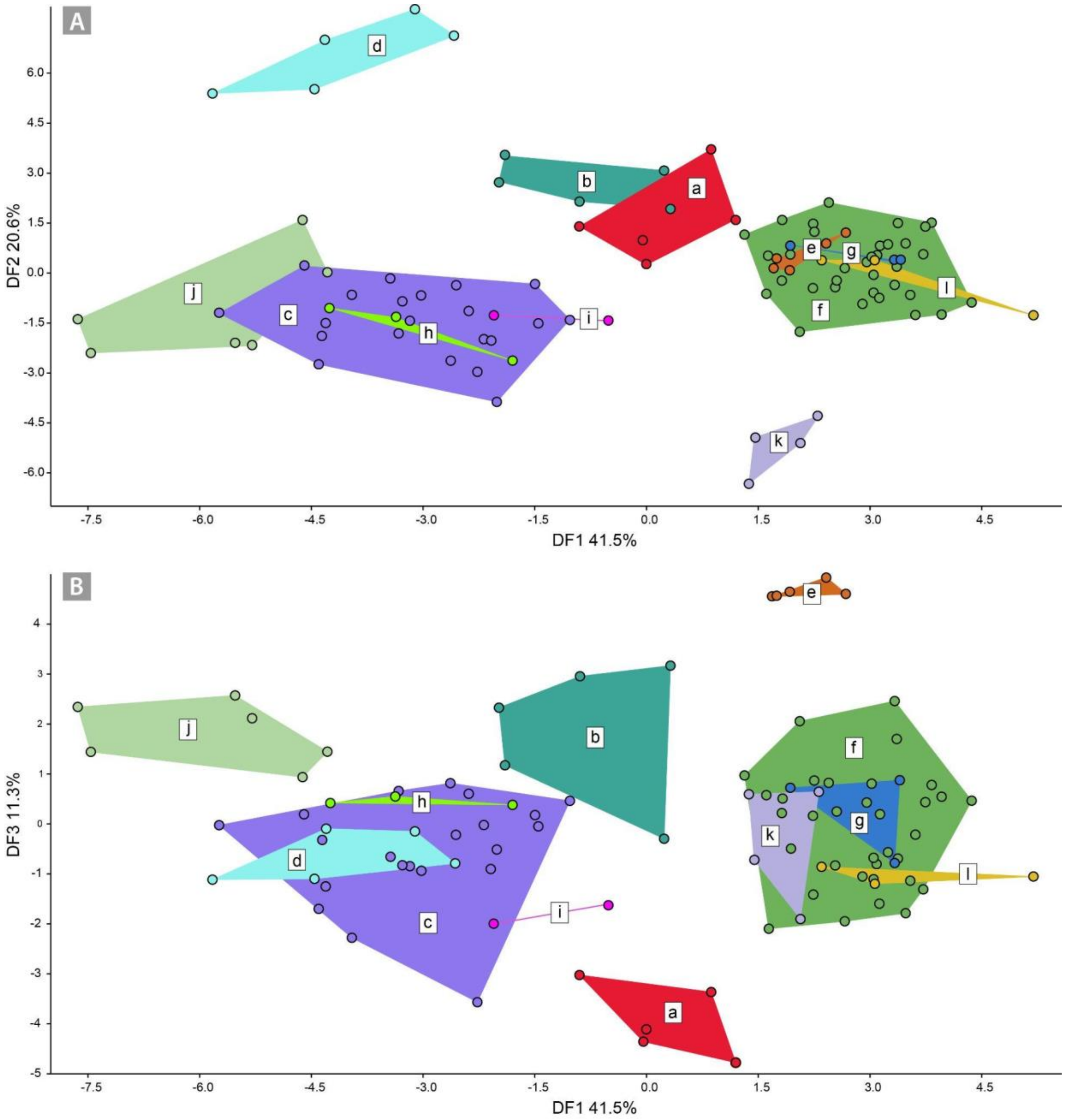

Figure S3. Canonical discriminant analysis (**A:** DF1 *vs.* DF2; **B:** DF1 *vs.* DF3) of 12 predefined groups (corresponding to AFLP clusters) based on individual plants and 18 morphological characters. Colours and letters correspond to AFLP clusters in Figure 1.

Table S1. Geographic origin and number of individuals of *Sorbus* populations included in the analyses of AFLP, nuclear microsatellite, plastid DNA sequencing, flow cytometric and morphometric data, respectively.

No.	ID	LOCALITY	*Sorbus aucuparia*	*Sorbus aria*	Subgen. *Soraria*: *Sorbus aria* × *S. austriaca*	Subgen. *Soraria*: *Sorbus austriaca*	Subgen. *Soraria*: *Sorbus mougeotii*	Subgen. *Soraria*: *Sorbus pauca*	Subgen. *Soraria*: *Sorbus pekarovae*	Subgen. *Soraria*: *Sorbus anglica*	Subgen. *Soraria*: *Sorbus cuneifolia*	TOTAL per locality
1	Shi	**Shija Gorge, Bicaj, Kukes, Albania,** 41° 05' 03" N, 20° 25' 56" E, 900 m; Leg. B. Frajman, P. Schönswetter & M. Falch (SARA 60022)	–, –, –, –, –	2, 2, –, –, –	–, –, –, –, –	–, –, –, –, –	–, –, –, –, –	–, –, –, –, –	–, –, –, –, –	–, –, –, –, –	–, –, –, –, –	**2, 2, –, –, –**
2	Suv	**Suva planina, Serbia,** 43° 09' 24" N, 22° 13' 17" E, 1566 m; Leg. M. Niketić (SARA 60027, GenBank OQ511841, OQ511875)	–, –, –, –, –	–, –, –, –, –	–, –, –, –, –	5, 5, 1, 3, 5	–, –, –, –, –	–, –, –, –, –	–, –, –, –, –	–, –, –, –, –	–, –, –, –, –	**5, 5, 1, 3, 5**
3	Bai	**Băile Herculane, Romania,** 44° 52′ 45″ N, 22° 25′ 58″ E, 936 m; Leg. Cs. Németh (Herbarium Cs. Németh 934/3, GenBank OQ511840, OQ511874)	–, –, –, –, –	–, –, –, –, –	–, –, –, –, –	3, 3, 1, –, 3	–, –, –, –, –	–, –, –, –, –	–, –, –, –, –	–, –, –, –, –	–, –, –, –, –	**3, 3, 1, –, 3**
4	Cic	**Ciclova Montană, Romania,** 45° 03′ 02″ N, 21° 46′ 05″ E, 821 m; Leg. Cs. Németh (Herbarium Cs. Németh 909/2, GenBank OQ511844, OQ511878)	–, –, –, –, –	–, –, –, –, –	–, –, –, –, –	3, 3, 1, –, 3	–, –, –, –, –	–, –, –, –, –	–, –, –, –, –	–, –, –, –, –	–, –, –, –, –	**3, 3, 1, –, 3**
5	Kop	**Kopaonik, Serbia,** 43° 17' 22" N, 20° 47' 36" E, 1630 m; Leg. A. Hajrudinović-Bogunić & F. Bogunić (SARA 60028)	2, 2, –, 2, –	–, –, –, –, –	–, –, –, –, –	–, –, –, –, –	–, –, –, –, –	–, –, –, –, –	–, –, –, –, –	–, –, –, –, –	–, –, –, –, –	**2, 2, –, 2, –**
6	Moj	**Mojstirska Draga, Serbia,** 42° 52' 26" N, 20° 24' 13" E, 1500 m; Leg. A. Hajrudinović-Bogunić & F. Bogunić (SARA 60029; SARA 60030, GenBank OQ511821, OQ511855)	–, –, –, –, –	1, 1, –, 1, –	2, 2, 1, 1, 2	–, –, –, –, –	–, –, –, –, –	–, –, –, –, –	–, –, –, –, –	–, –, –, –, –	–, –, –, –, –	**3, 3, 1, 2, 2**
7	Rug	**Rugovska klisura, Kosovo,** 42° 39' 41" N, 20° 14' 36" E, 580 m; Leg. B. Frajman, P. Schönswetter & M. Falch (SARA 60024; SARA 60025; SARA 60026, GenBank OQ511848, OQ511882)	–, –, –, –, –	1, 1, –, –, –	1, 1, –, –, 1	2, 2, 1, –, 2	–, –, –, –, –	–, –, –, –, –	–, –, –, –, –	–, –, –, –, –	–, –, –, –, –	**4, 4, 1, –, 3**
8	Uga	**Ugao, Serbia,** 43° 02' 40" N, 20° 05' 08" E, 1290 m; Leg. A. Hajrudinović-Bogunić & F. Bogunić (no voucher; SARA 60031, GenBank OQ511847, OQ511881)	–, –, –, –, –	1, 1, –, –, –	–, –, –, –, –	4, 4, 1, 1, 4	–, –, –, –, –	–, –, –, –, –	–, –, –, –, –	–, –, –, –, –	–, –, –, –, –	**5, 5, 1, 1, 4**
9	Zla	**Mt. Zlatar, Serbia,** 43° 25' 12" N, 19° 49' 33" E, 1200 m; Leg. A. Hajrudinović-Bogunić & F. Bogunić (SARA 60032, GenBank OQ511838, OQ511872)	–, –, –, –, –	–, –, –, –, –	–, –, –, –, –	5, 5, 1, 1, 5	–, –, –, –, –	–, –, –, –, –	–, –, –, –, –	–, –, –, –, –	–, –, –, –, –	**5, 5, 1, 1, 5**
10	Ora	**Orahovac, Bijela gora, Bosnia and Herzegovina,** 42° 40' 28" N, 18° 30' 28" E, 854 m; Leg. B. Frajman, P. Schönswetter & M. Falch (SARA 53262)	–, –, –, –, –	2, 2, –, –, –	–, –, –, –, –	–, –, –, –, –	–, –, –, –, –	–, –, –, –, –	–, –, –, –, –	–, –, –, –, –	–, –, –, –, –	**2, 2, –, –, –**
11	Vrb	**Vrba, Bosnia and Herzegovina,** 43° 10' 52" N, 18° 34' 06" E, 1090 m; Leg. A. Hajrudinović-Bogunić & F. Bogunić (SARA 51411, GenBank KP794828, KP794854, GenBank OQ511850, OQ511884)	–, –, –, –, –	–, –, –, –, –	–, –, –, –, –	5, 5, 2, 5, 5	–, –, –, –, –	–, –, –, –, –	–, –, –, –, –	–, –, –, –, –	–, –, –, –, –	**5, 5, 2, 5, 5**
12	Mag	**Mt. Maglić, Bosnia and Herzegovina,** 43° 19' 09" N, 18° 42' 47" E, 1304 m; Leg. B. Frajman, P. Schönswetter & M. Falch (SARA 53263, GenBank OQ511834, OQ511868)	–, –, –, –, –	–, –, –, –, –	–, –, –, –, –	5, 5, 1, –, 5	–, –, –, –, –	–, –, –, –, –	–, –, –, –, –	–, –, –, –, –	–, –, –, –, –	**5, 5, 1, –, 5**
13	Kal	**Kalinovik, Bosnia and Herzegovina,** 43° 31' 09" N, 18° 26' 50" E, 1134 m; Leg. A. Medić (SARA 53264, GenBank OQ511835, OQ511869)	–, –, –, –, –	–, –, –, –, –	–, –, –, –, –	5, 5, 1, 3, 5	–, –, –, –, –	–, –, –, –, –	–, –, –, –, –	–, –, –, –, –	–, –, –, –, –	**5, 5, 1, 3, 5**
14	Igm	**Mt. Igman, Bosnia and Herzegovina,** 43° 44' 21" N, 18° 17' 54" E, 1380 m; Leg. A. Hajrudinović-Bogunić & F. Bogunić (SARA 51417, GenBank KP794823, KP794849; SARA 51416, GenBank KP794813, KP794839, GenBank KP794815, KP794841, GenBank KP794816, KP794842; SARA 51418, GenBank KP794825, KP794851, GenBank OQ511833, OQ511867)	3, 3, 1, 3, –	3, 3, 3, 3, –	–, –, –, –, –	7, 7, 2, 7, 7	–, –, –, –, –	–, –, –, –, –	–, –, –, –, –	–, –, –, –, –	–, –, –, –, –	**13, 13, 6, 13, 7**
15	Bli	**Blidinje lake, Bosnia and Herzegovina,** 43° 36' 38" N, 17° 31' 09" E, 1180 m; Leg. A. Hajrudinović-Bogunić & F. Bogunić (SARA 53265, GenBank OQ511829, OQ511863)	–, –, –, –, –	–, –, –, –, –	–, –, –, –, –	5, 5, 1, 5, 5	–, –, –, –, –	–, –, –, –, –	–, –, –, –, –	–, –, –, –, –	–, –, –, –, –	**5, 5, 1, 5, 5**
16	Gra	**Gradac, Bosnia and Herzegovina,** 43° 25' 56" N, 17° 23' 27" E, 1200 m; Leg. A. Hajrudinović-Bogunić & F. Bogunić (SARA 53266)	–, –, –, –, –	3, 3, –, 3, –	–, –, –, –, –	–, –, –, –, –	–, –, –, –, –	–, –, –, –, –	–, –, –, –, –	–, –, –, –, –	–, –, –, –, –	**3, 3, –, 3, –**
17	Bio	**Mt. Biokovo, Croatia,** 43° 19' 09" N, 17° 03' 16" E, 1400 m; Leg. A. Hajrudinović-Bogunić & F. Bogunić (SARA 60033; SARA 60034, GenBank OQ511830, OQ511864)	–, –, –, –, –	2, 2, –, 2, –	–, –, –, –, –	5, 5, 1, 3, 5	–, –, –, –, –	–, –, –, –, –	–, –, –, –, –	–, –, –, –, –	–, –, –, –, –	**6, 6, 1, 4, 5**
18	Bas	**Baške Oštarije, Mt. Velebit, Croatia,** 44° 31' 45" N, 15° 11' 11" E, 945 m; Leg. A. Hajrudinović-Bogunić & F. Bogunić (SARA 60035; SARA 60036, GenBank OQ511828, OQ511862)	2, 2, –, –, –	–, –, –, –, –	–, –, –, –, –	5, 5, 1, –, 5	–, –, –, –, –	–, –, –, –, –	–, –, –, –, –	–, –, –, –, –	–, –, –, –, –	**7, 7, 1, –, 5**
19	Tur	**Turček, Slovakia,** 48° 44′ 39″ N, 18° 54′ 47″ E, 790 m; Leg. C. Németh (Herbarium Cs. Németh 4013/2, GenBank OQ511849, OQ511883)	–, –, –, –, –	–, –, –, –, –	–, –, –, –, –	2, 2, 1, –, 2	–, –, –, –, –	–, –, –, –, –	–, –, –, –, –	–, –, –, –, –	–, –, –, –, –	**2, 2, 1, –, 2**
20	Sin	**Siná, Slovakia,** 49° 00′ 00″ N, 19° 34′ 18″ E, 1150 m; Leg. V. Kučerová (no voucher)	–, –, –, –, –	–, –, –, –, –	–, –, –, –, –	3, 3, –, –, 3	–, –, –, –, –	–, –, –, –, –	–, –, –, –, –	–, –, –, –, –	–, –, –, –, –	**3, 3, –, –, 3**
21	Pek	**Pekárová, Slovakia,** 48° 57′ 25″ N, 18° 57′ 51″ E, 880 m; Leg. V. Kučerová (no voucher, GenBank OQ511837, OQ511871)	–, –, –, –, –	–, –, –, –, –	–, –, –, –, –	–, –, –, –, –	–, –, –, –, –	–, –, –, –, –	4, 4, 1, –, 4	–, –, –, –, –	–, –, –, –, –	**4, 4, 1, –, 4**
22	Bez	**Bezděz hill, Česká Lípa, N Bohemia, Czech Republic,** 50° 32' 23" N, 14° 43' 18" E, 580 m; Leg. M. Lepší & P. Lepší, (CB 79599, GenBank OQ511819, OQ511853)	–, –, –, –, –	–, –, –, –, –		–, –, –, –, –	–, –, –, –, –	1, 1, 1, –, –	–, –, –, –, –	–, –, –, –, –	–, –, –, –, –	**1, 1, 1, –, –**
23	Fal	**Fallenstein, Nordostliche Kalkalpen, Austria,** 47° 44' 06" N, 15° 20' 16" E, 940 m; Leg. W. Gutermann, D. Reich, R. Sander, M. Hofbauer, C. Gilli, B. Weis & M. Thalinger (SARA 60040; SARA 60041; SARA 60042, GenBank OQ511823, OQ511857, GenBank OQ511824, OQ511858)	2, 2, –, –, –	3, 3, –, –, –	–, –, –, –, –	5, 5, 2, –, 5	–, –, –, –, –	–, –, –, –, –	–, –, –, –, –	–, –, –, –, –	–, –, –, –, –	**10, 10, 2, –, 5**
24	Ker	**Kerngraben, Altenberg an der Rax, Steirermark, Austria,** 47° 41' 11" N, 15° 39' 19" E, 950 m; Leg. A. Tribsch (SARA 60043, GenBank OQ511831, OQ511865)	–, –, –, –, –	–, –, –, –, –	–, –, –, –, –	5, 5, 1, –, 5	–, –, –, –, –	–, –, –, –, –	–, –, –, –, –	–, –, –, –, –	–, –, –, –, –	**5, 5, 1, –, 5**
25	Kar	**Karavanke, Slovenia,** 46° 27' 59" N, 13° 59' 26" E, 1050 m; Leg. R. Brus (SARA 60037, GenBank OQ511832, OQ511866)	–, –, –, –, –	–, –, –, –, –	–, –, –, –, –	5, 5, 1, –, 5	–, –, –, –, –	–, –, –, –, –	–, –, –, –, –	–, –, –, –, –	–, –, –, –, –	**5, 5, 1, –, 5**
26	Dob	**Dobratsch, Gailtaler Alpen, Austria,** 46° 35' 28" N, 13° 43' 43" E, 1500 m; Leg. B. Frajman & P. Schönswetter (SARA 60044; SARA 60045; SARA 60046, GenBank OQ511836, OQ511870)	2, 2, –, –, –	2, 2, –, –, –	–, –, –, –, –	5, 5, 1, –, 5	–, –, –, –, –	–, –, –, –, –	–, –, –, –, –	–, –, –, –, –	–, –, –, –, –	**9, 9, 1, –, 5**
27	Sch	**Schafberg bei St. Wolfgang, Flachgau, Salzburg, Austria,** 47° 46' 23" N, 13° 26' 26" E, 1650 m; Leg. P. Pilsl (Herbarium Peter Pilsl 19097, GenBank OQ511843, OQ511877, GenBank OQ511845, OQ511879)	–, –, –, –, –	–, –, –, –, –	–, –, –, –, –	3, 3, 2, –, –	–, –, –, –, –	–, –, –, –, –	–, –, –, –, –	–, –, –, –, –	–, –, –, –, –	**3, 3, 2, –, –**
28	Bre	**Breitenstein, Bayerische Voralpen, Germany,** 47° 43' 55" N, 12° 00' 55" E, 920 m; Leg. P. Schönswetter (SARA 60038; SARA 60039, GenBank OQ511822, OQ511856)	2, 2, –, –, –	7, 7, 1, –, –	–, –, –, –, –	–, –, –, –, –	–, –, –, –, –	–, –, –, –, –	–, –, –, –, –	–, –, –, –, –	–, –, –, –, –	**9, 9, 1, –, –**
29	Nor	**Nordkette, Karwendel, Tirol, Austria,** 47° 16' 41" N, 11° 20' 49" E, 970 m; Leg. B. Frajman & P. Schönswetter (SARA 60047)	–, –, –, –, –	3, 3, –, –, –	–, –, –, –, –	–, –, –, –, –	–, –, –, –, –	–, –, –, –, –	–, –, –, –, –	–, –, –, –, –	–, –, –, –, –	**3, 3, –, –, –**
30	Mie	**Mieminger Gebirge, Tirol, Austria,** 47° 19' 39" N, 10° 57' 58" E, 1100 m; Leg. M. Falch (SARA 60048, GenBank OQ511820, OQ511854)	–, –, –, –, –	5, 5, 1, –, –	–, –, –, –, –	–, –, –, –, –	–, –, –, –, –	–, –, –, –, –	–, –, –, –, –	–, –, –, –, –	–, –, –, –, –	**5, 5, 1, –, –**
31	Sta	**Stanzertal, Vorarlberg, Austria,** 47° 08' 53" N, 10° 19' 07" E, 1320 m; Leg. P. Schönswetter (SARA 60049; SARA 60050; SARA 60051, GenBank OQ511825, OQ511859)	2, 2, –, –, –	2, 2, –, –, –	–, –, –, –, –	–, –, –, –, –	3, 3, 1, –, 3	–, –, –, –, –	–, –, –, –, –	–, –, –, –, –	–, –, –, –, –	**7, 7, 1, –, 3**
32	Mon	**Schruns, Montafon, Vorarlberg, Austria,** 47° 06' 02" N, 09° 56' 57" E, 1120 m; Leg. P. Schönswetter & B. Frajman (SARA 60052; SARA 60053, GenBank OQ511818, OQ511852)	2, 2, –, –, –	6, 6, 1, –, –	–, –, –, –, –	–, –, –, –, –	–, –, –, –, –	–, –, –, –, –	–, –, –, –, –	–, –, –, –, –	–, –, –, –, –	**8, 8, 1, –, –**
33	Uit	**Uitikon, Zurich, Switzerland,** 47° 21' 55" N, 08° 27' 49" E, 630 m; Leg. F. Gugerli (SARA 60054, GenBank OQ511827, OQ511861)	–, –, –, –, –	–, –, –, –, –	–, –, –, –, –	–, –, –, –, –	1, 1, 1, –, 1	–, –, –, –, –	–, –, –, –, –	–, –, –, –, –	–, –, –, –, –	**1, 1, 1, –, 1**
34	Wal	**Walliser Alpen, Switzerland,** 46° 01' 57" N, 07° 06' 03" E, 1540 m; Leg. C. Pachschwoll & J. Theurillat (no voucher, GenBank OQ511846, OQ511880)	–, –, –, –, –	–, –, –, –, –	–, –, –, –, –	–, –, –, –, –	1, 1, 1, –, 1	–, –, –, –, –	–, –, –, –, –	–, –, –, –, –	–, –, –, –, –	**1, 1, 1, –, 1**

**35**	**Pob**	**Poblet, Spain,** 41° 21' 56" N, 01° 04' 48" E, 830 m; Leg. J. Vallès (SARA 60055)	–, –, –, –, –	3, 2, –, 3, –	–, –, –, –, –	–, –, –, –, –	–, –, –, –, –	–, –, –, –, –	–, –, –, –, –	–, –, –, –, –	–, –, –, –, –	**3, 2, –, 3, –**
**36**	**Shp**	**Ship Rock, Coldwell Rocks, Symonds Yat, Gloucestershire, England, UK,** 51° 50' 18" N, 02° 37' 27" W, 120m; Leg. T. Rich (NBGW 1341a,b; NBGW 1384a,b, GenBank OQ511826, OQ511860)	–, –, –, –, –	–, –, –, –, –	–, –, –, –, –	–, –, –, –, –	–, –, –, –, –	–, –, –, –, –	–, –, –, –, –	5, 5, 1, –, –	–, –, –, –, –	**5, 5, 1, –, –**
**37**	**Cre**	**Creigiau Eglwyseg, Denbighshire, Wales, UK,** 52° 59' 53" N, 03° 09' 50" W, 380m; Leg. T. Knight (cultivated at NBGW, GenBank OQ511839, OQ511873)	–, –, –, –, –	–, –, –, –, –	–, –, –, –, –	–, –, –, –, –	–, –, –, –, –	–, –, –, –, –	–, –, –, –, –	–, –, –, –, –	5, 5, 1, –, –	**5, 5, 1, –, –**
**38***	**Skr**	**Mali i Tomorit, Skrapar, Albania,** 40° 37' 12" N, 20° 11' 31" E, 1542 m (SARA 60023, GenBank OQ511817, OQ511851)	–, –, –, –, –	–, –, 1, –, –	–, –, –, –, –	–, –, –, –, –	–, –, –, –, –	–, –, –, –, –	–, –, –, –, –	–, –, –, –, –	–, –, –, –, –	**–, –, 1, –, –**
**39***	**Umo**	**Umoljani, Mt. Bjelašnica, Bosnia and Herzegovina,** 43° 39′ 31″ N, 18° 13′ 49″ E, 1260m; Leg. A. Hajrudinović, F. Bogunić (SARA 51413, GenBank KP794814, KP794840, GenBank KP794817, KP794843; SARA 51414, GenBank KP794827, KP794853)	–, –, –, –, –	–, –, 2, –, –	–, –, –, –, –	–, –, 1, –, –	–, –, –, –, –	–, –, –, –, –	–, –, –, –, –	–, –, –, –, –	–, –, –, –, –	**–, –, 3, –, –**
**40***	**Kru**	**Mt. Krug planina, Bosnia and Herzegovina,** 43° 50' 32" N, 17° 11' 59" E, 1300m; Leg. A. Hajrudinović, F. Bogunić (SARA 51408, GenBank KP794824, KP794850; SARA 51406, GenBank KP794811, KP794837, GenBank KP794812, KP794838; SARA 51409, GenBank KP794826, KP794852)	–, –, 1, –, –	–, –, 2, –, –	–, –, –, –, –	–, –, 1, –, –	–, –, –, –, –	–, –, –, –, –	–, –, –, –, –	–, –, –, –, –	–, –, –, –, –	**–, –, 4, –, –**
**41***	**Slo**	**Mt. Slovinj, Bosnia and Herzegovina,** 43° 59' 41" N, 16° 59' 06" E, 1350 m; Leg. A. Hajrudinović, F. Bogunić (SARA 51410, GenBank KP794809, KP794835)	–, –, –, –, –	–, –, 1, –, –	–, –, –, –, –	–, –, –, –, –	–, –, –, –, –	–, –, –, –, –	–, –, –, –, –	–, –, –, –, –	–, –, –, –, –	**–, –, 1, –, –**
**42***	**Hai**	**Hainburg, Niederösterreich, Austria** (FC382 from Pellicer *et al*. 2012, GenBank KP794807, KP794833)	–, –, –, –, –	–, –, 1, –, –	–, –, –, –, –	–, –, –, –, –	–, –, –, –, –	–, –, –, –, –	–, –, –, –, –	–, –, –, –, –	–, –, –, –, –	**–, –, 1, –, –**
**43***	**Fus**	**Fuschlsee, Salzburg, Austria,** 47° 48' 54" N, 13° 15' 14" E, 665 m; Leg. A. Tribsch (AT 112316, GenBank OQ511842, OQ511876)	–, –, –, –, –	–, –, –, –, –	–, –, –, –, –	–, –, 1, –, –	–, –, –, –, –	–, –, –, –, –	–, –, –, –, –	–, –, –, –, –	–, –, –, –, –	**–, –, 1, –, –**
**44***	**Inn**	**Innsbruck, Tirol, Austria,** 47° 16' 37" N, 11° 22' 35" E, 900 m; Leg. P. Schönswetter, B. Frajman, A. Hajrudinović (no vouchers; GenBank KP794818, KP794844; GenBank KP794806, KP794832)	–, –, 1, –, –	–, –, 1, –, –	–, –, –, –, –	–, –, –, –, –	–, –, –, –, –	–, –, –, –, –	–, –, –, –, –	–, –, –, –, –	–, –, –, –, –	**–, –, 2, –, –**
**45***	**Pla**	**The Plain, Leigh Woods, England, UK** (FC007 from Pellicer *et al*. 2012, GenBank KP794820, KP794846)	–, –, 1, –, –	–, –, –, –, –	–, –, –, –, –	–, –, –, –, –	–, –, –, –, –	–, –, –, –, –	–, –, –, –, –	–, –, –, –, –	–, –, –, –, –	**–, –, 1, –, –**
**46***	**Off**	**Offa´s Dyke, Tidenham Chase, Wales, UK** (FC154 from Pellicer *et al*. 2012, GenBank KP794808, KP794834)	–, –, –, –, –	–, –, 1, –, –	–, –, –, –, –	–, –, –, –, –	–, –, –, –, –	–, –, –, –, –	–, –, –, –, –	–, –, –, –, –	–, –, –, –, –	**–, –, 1, –, –**
**47***	**Uus**	**Uusimaa, Vihti, Finland** (FC370 from Pellicer *et al*. 2012, GenBank KP794821, KP794847)	–, –, 1, –, –	–, –, –, –, –	–, –, –, –, –	–, –, –, –, –	–, –, –, –, –	–, –, –, –, –	–, –, –, –, –	–, –, –, –, –	–, –, –, –, –	**–, –, 1, –, –**
		**TOTAL per taxon**	**17, 17, 5, 5, –**	**45, 44, 15, 11, –**	**3, 3, 1, 1, 3**	**87, 87, 26, 28, 84**	**5, 5, 3, –, 5**	**1, 1, 1, –, –**	**4, 4, 1, –, 4**	**5, 5, 1, –, –**	**5, 5, 1, –, –**	**172, 171, 54, 45, 96**

Additional localities used only in plastid DNA analysis; samples from 39 to 47* are published in Hajrudinović *et al*. (2015*a*).

Table S2. Indices of population clonal diversity based on nuclear microsatellite data for the studied populations of *Sorbus* subgen. *Soraria*.

**Nb.**	**Locality No.**	**Number of individuals sampled (*N*)**	**Number of multilocus genotypes detected per locality (*Ng*)**	**Effective number of genotypes (*Eff*)**	**Genotypic diversity (*Div*)**
1.	**2**	5	1	1	0
2.	**3**	3	1	1	0
3.	**4**	3	1	1	0
4.	**6**	2	1	1	0
5.	**7**	3	3	3	1
6.	**8**	4	2	2	0.67
7.	**9**	5	1	1	0
8.	**11**	5	2	1.47	0.4
9.	**12**	5	1	1	0
10.	**13**	5	1	1	0
11.	**14**	7	3	2.33	0.67
12.	**15**	5	3	2.78	0.8
13.	**17**	5	1	1	0
14.	**18**	5	1	1	0
15.	**19**	2	1	1	0
16.	**20**	3	1	1	0
17.	**21**	4	1	1	0
18.	**22**	1	1	1	/
19.	**23**	5	1	1	0
20.	**24**	5	1	1	0
21.	**25**	5	1	1	0
22.	**26**	5	1	1	0
23.	**27**	3	1	1	0
24.	**31**	3	1	1	0
25.	**33**	1	1	1	/
26.	**34**	1	1	1	/
27.	**36**	5	1	1	0
28.	**37**	5	2	1.92	0.60
		**110**			

Table S3. Allele composition in six nuclear microsatellite loci for the presumed parental taxa of *Sorbus* subgen. *Soraria*.

**Taxon**	**Ploidy***	**MSS13**	**MSS5**	**CH01F02**	**D11**	**MSS16**	**H10**	***N* of individuals**	***N* of localities**
S. aria	2*x*	193, 191, 195, 197, 199, 201, 207, 211	120, 110, 114, 116, 118, 122, 124, 126, 130, 136, 138, 140	190, 194, 196, 198, 200, 202, 214	137, 139, 145, 147, 149, 151, 153, 155, 157, 161, 167, 173, 175, 177, 179, 181, 185, 187, 189, 195, 199, 205	154, 156, 158, 160, 162, 164, 166	78, 80, 82, 84, 88, 90, 92, 96	24	9
S. aria	3*x*	189, 193, 195, 197, 199, 211	110, 112, 120, 122, 124, 126, 128, 130, 134, 150	190, 192, 196, 198, 200, 202, 214	137, 147, 149, 151, 153, 157, 161, 163, 165, 173, 177, 179, 181, 201, 203, 215	154, 158, 160, 162. 164, 166	78, 80, 82, 88. 90, 92, 104, 110	13	7
S. aria	4*x*	187, 191, 193, 195, 197, 199, 201, 203	110, 112, 114, 116, 118, 122, 124, 128, 130, 132	190, 196, 198, 200, 202, 204, 215, 216	137, 149, 151, 153, 169, 175, 179, 185, 205	154, 156, 158, 160, 162, 166, 170	78, 82, 90, 92	7	6
S. aucuparia	2*x*	181, 183, 185, 187, 189, 191, 193, 195	98, 96, 97, 99, 100, 114, 126, 130	186, 188	131, 139, 141, 145, 147, 149, 157	154, 156, 158	86, 88, 90, 92, 94, 96, 98, 102, 104, 106	17	8
							**Total**	**61**	

*estimated from nuclear microsatellites.

Table S4. Results of principal component analysis (PCA, see Figure S2) and canonical discriminant analysis (CDA, see Figure S3) of *Soraria* individuals based on the morphological characters of leaves.

**Leaf character**	**PCA eigenvectors**		**CDA total canonical structure values**	
	**PC 1**	**PC 2**	**DF 1**	**DF 1**
LLEAV	0.25	0.14	-0.09	-0.12
LPET	0.18	0.13	-0.08	-0.12
1SEINL	0.21	-0.09	-0.26	0.11
1NERV	0.28	-0.31	**-0.37**	-0.15
2SEINL	0.29	0.02	-0.19	-0.11
2NERV	**0.34**	-0.17	**-0.33**	-0.32
3SEINL	0.28	0.09	-0.20	-0.13
3NERV	**0.34**	-0.06	-0.29	**-0.47**
WLEAV	**0.35**	0.02	-0.31	**-0.62**
MXWLEAV	0.16	0.10	0.01	-0.30
1NANG	0.17	**0.36**	-0.06	-0.29
2NANG	0.07	**0.47**	0.09	-0.32
3NANG	0.03	**0.49**	0.07	0.10
1ALEAV	0.24	-0.10	-0.26	-0.27
1BLEAV	0.11	0.18	-0.01	-0.19
NNER	0.00	**0.41**	**0.35**	**-0.52**
LLEAV / WLEAV	-0.28	0.08	**0.52**	**0.54**
WLEAV / MXLEAV	0.22	-0.06	0.05	-0.10

Table S5. Classification matrix with Jackknife procedure (82% of cases correctly classified) for the dataset of analysed *Sorbus* individuals based on morphometric leaf measurements (groups are defined according to AFLP clusters, Figure 1B).

**AFLP group**	**d**	**j**	**f**	**c**	**g**	**i**	**h**	**k**	**b**	**e**	**a**	**l**	**Individuals in predefined AFLP groups**	**Number of correctly classified individuals**
**d**	**5**	0	0	0	0	0	0	0	0	0	0	0	**5**	**5**
**j**	0	**5**	0	0	0	0	1	0	0	0	0	0	**6**	**5**
**f**	0	0	**28**	0	2	0	0	0	0	1	1	2	**34**	**28**
**c**	0	0	0	**19**	0	1	1	0	0	0	0	0	**21**	**19**
**g**	0	0	0	0	**3**	0	0	0	0	0	0	0	**3**	**3**
**i**	0	0	0	0	0	**1**	1	0	0	0	0	0	**2**	**1**
**h**	0	1	0	0	0	1	**1**	0	0	0	0	0	**3**	**1**
**k**	0	0	0	0	0	0	0	**4**	0	0	0	0	**4**	**4**
**b**	0	0	0	0	0	0	0	0	**4**	0	1	0	**5**	**4**
**e**	0	0	0	0	0	0	0	0	0	**5**	0	0	**5**	**5**
**a**	0	0	0	0	0	0	0	0	1	0	**4**	0	**5**	**4**
**l**	0	0	2	0	0	0	0	0	0	1	0	**0**	**3**	**0**
**Individuals in *a posteriori* classified groups**	**5**	**6**	**30**	**19**	**5**	**3**	**4**	**4**	**5**	**7**	**6**	**2**	**96**	**79 (82%)**

Table S6. Classification matrix with Jackknife procedure (88% of cases correctly classified) for the dataset of analysed *Sorbus austriaca* individuals based on morphometric leaf measurements (groups are defined according to AFLP clusters, Figure 1B).

AFLP group	d	j	f	c	g	i	h	b	e	Individuals in predefined AFLP groups	Number of correctly classified individuals
**d**	**5**	0	0	0	0	0	0	0	0	**5**	**5**
**j**	0	**5**	0	0	0	0	1	0	0	**6**	**5**
**f**	0	0	**31**	0	2	1	0	0	0	**34**	**31**
**c**	0	0	0	**18**	0	1	1	1	0	**21**	**18**
**g**	0	0	0	0	**3**	0	0	0	0	**3**	**3**
**i**	0	0	0	0	0	**2**	0	0	0	**2**	**2**
**h**	0	1	0	0	0	1	**1**	0	0	**3**	**1**
**b**	0	0	1	0	0	0	0	**4**	0	**5**	**4**
**e**	0	0	0	0	0	0	0	0	**5**	**5**	**5**
**Individuals in *a posteriori* classified groups**	**5**	**6**	**32**	**18**	**5**	**5**	**3**	**5**	**5**	**84**	**74 (88%)**

Table S7. Descriptive statistics of leaf morphometrics for *Sorbus* subgen. *Soraria* individuals presented for AFLP groups.

AFLP cluster	Taxon	Group count	Descriptive statistics	LLEAV	LPET	1SEINL	1NERV	2SEINL	2NERV	3SEINL	3NERV	WLEAV	MXWLEAV	1NANG	2NANG	3NANG	1ALEAV	1BLEAV	NNER
a	*S. mougeotii*	5	**Ar. mean**	**74.5**	**11.9**	**2.3**	**28.4**	**3.0**	**33.2**	**4.0**	**34.6**	**47.5**	**41.8**	**54.2**	**45.1**	**39.2**	**28.6**	**22.8**	**7.5**
			SD	9.6	4.2	1.3	2.5	1.2	5.0	1.8	3.9	6.8	5.0	4.9	3.8	3.7	5.6	5.1	1.1
			Min	63.3	6.0	1.0	24.0	2.0	26.0	2.3	31.0	41.7	33.3	48.3	40.7	35.0	23.0	16.0	6.3
			Max	84.3	15.3	3.7	30.0	4.3	38.0	6.0	39.0	55.0	45.0	60.0	50.0	42.0	34.7	30.3	8.7
			CV%	12.9	35.0	56.2	8.8	40.0	14.9	43.5	11.3	14.2	12.0	9.0	8.4	9.4	19.7	22.6	14.0
b	*S. austriaca*	5	**Ar. mean**	**77.8**	**14.6**	**6.4**	**28.4**	**5.9**	**31.8**	**5.0**	**32.9**	**49.3**	**33.9**	**54.4**	**50.8**	**42.1**	**24.7**	**29.5**	**8.0**
			SD	11.2	4.2	4.1	3.5	2.2	3.5	1.2	3.1	5.0	2.4	3.0	6.2	3.1	3.3	6.0	0.7
			Min	65.0	7.5	2.5	25.3	3.0	29.0	4.0	27.5	43.0	31.3	51.3	43.5	37.5	19.5	21.5	7.0
			Max	96.0	18.0	12.5	34.0	8.0	37.5	7.0	35.5	54.5	37.0	58.3	58.0	45.0	28.0	37.7	8.7
			CV%	14.4	28.6	64.5	12.3	37.7	11.1	24.9	9.5	10.1	7.1	5.5	12.1	7.3	13.2	20.4	9.0
c	*S. austriaca*	21	**Ar. mean**	**90.1**	**18.0**	**3.4**	**28.0**	**5.4**	**37.4**	**5.9**	**39.6**	**57.8**	**45.1**	**57.2**	**50.2**	**45.3**	**25.8**	**30.9**	**10.4**
			SD	4.6	3.5	1.3	3.4	1.6	4.2	1.6	3.6	6.5	4.9	2.5	2.4	3.2	4.2	5.9	0.8
			Min	78.7	11.0	1.0	23.2	3.2	29.0	3.0	33.7	41.0	35.0	52.3	45.7	37.7	16.0	23.0	9.0
			Max	97.0	23.0	7.0	36.0	10.0	47.0	9.0	48.0	70.0	53.8	61.0	55.1	54.0	35.0	44.0	12.0
			CV%	5.1	19.3	37.2	12.2	29.8	11.2	26.3	9.1	11.2	10.8	4.3	4.7	7.1	16.4	19.1	7.4
d	*S. austriaca*	5	**Ar. mean**	**70.0**	**9.0**	**3.2**	**25.6**	**4.3**	**29.6**	**3.7**	**28.6**	**38.8**	**33.4**	**44.8**	**41.8**	**39.8**	**20.0**	**22.4**	**7.6**
			SD	4.8	0.0	1.0	3.7	2.1	3.1	1.3	2.9	2.3	2.9	6.6	7.1	6.5	3.5	5.3	0.9
			Min	62.0	9.0	2.0	21.0	3.0	25.0	2.5	24.0	36.0	30.0	40.0	36.0	32.0	14.0	15.0	7.0
			Max	75.0	9.0	4.5	31.0	8.0	33.0	5.0	31.0	42.0	37.0	56.0	54.0	50.0	22.0	30.0	9.0
			CV%	6.9	0.0	32.4	14.5	49.1	10.6	33.9	10.1	5.9	8.6	14.8	17.0	16.3	17.3	23.7	11.8
e	*S. austriaca*	5	**Ar. mean**	**82.2**	**17.0**	**3.4**	**35.5**	**5.5**	**41.0**	**7.4**	**41.0**	**61.0**	**37.5**	**51.0**	**49.5**	**42.0**	**23.8**	**28.8**	**8.6**
			SD	0.7	0.7	0.3	0.4	0.4	0.7	0.3	0.7	0.7	0.4	0.7	0.4	0.7	0.5	0.5	0.5
			Min	81.0	16.0	3.0	35.0	5.0	40.0	7.0	40.0	60.0	37.0	50.0	49.0	41.0	23.0	28.0	8.0
			Max	83.0	18.0	3.7	36.0	6.0	42.0	7.7	42.0	62.0	38.0	52.0	50.0	43.0	24.5	29.5	9.0
			CV%	0.9	4.2	7.5	1.0	6.4	1.7	3.4	1.7	1.2	0.9	1.4	0.7	1.7	2.3	1.9	6.4
f	*S. austriaca*	34	**Ar. mean**	**84.4**	**16.2**	**4.8**	**37.9**	**6.3**	**44.3**	**5.8**	**44.0**	**62.9**	**40.8**	**51.4**	**43.6**	**37.4**	**31.3**	**28.7**	**7.8**
			SD	8.5	3.0	1.4	3.9	1.6	4.0	1.0	4.3	6.1	5.5	4.7	3.6	4.1	2.7	5.3	0.6
			Min	61.0	8.0	2.5	27.0	2.5	36.0	3.5	33.5	50.0	31.0	41.0	33.5	27.5	24.8	18.0	6.5
			Max	109	21.2	10.0	46.0	11.0	55.0	7.5	55.6	81.0	58.0	62.0	52.0	47.2	38.2	42.4	8.8
			CV%	10.0	18.8	29.5	10.2	25.2	9.1	17.6	9.8	9.8	13.6	9.1	8.3	10.9	8.6	18.3	7.7
g	*S. austriaca*	3	**Ar. mean**	**91.3**	**21.4**	**5.2**	**38.8**	**7.2**	**46.3**	**7.0**	**48.5**	**61.8**	**49.7**	**49.5**	**43.1**	**37.1**	**31.1**	**17.1**	**9.0**
			SD	0.8	1.6	0.6	2.6	1.3	3.7	0.6	3.1	2.6	2.8	0.4	1.2	1.4	1.4	2.1	0.0
			Min	90.4	19.8	4.7	36.0	6.1	42.0	6.5	45.0	60.0	46.6	49.2	42.0	36.2	29.6	14.8	9.0
			Max	91.8	23.0	5.9	41.2	8.7	48.6	7.7	50.8	64.8	52.2	50.0	44.4	38.8	32.4	19.0	9.0
			CV%	0.9	7.5	11.7	6.8	18.4	8.0	8.7	6.4	4.2	5.7	0.8	2.8	3.9	4.6	12.5	0.0
h	*S. austriaca*	3	**Ar. mean**	**88.7**	**11.0**	**1.8**	**30.0**	**3.8**	**38.0**	**4.2**	**43.0**	**59.3**	**50.3**	**46.0**	**46.7**	**42.0**	**25.7**	**24.0**	**10.0**
			SD	3.2	1.0	0.3	2.6	1.4	2.6	0.8	2.6	3.1	3.8	2.6	2.3	1.0	2.3	2.6	0.0
			Min	85.0	10.0	1.5	27.0	3.0	35.0	3.5	40.0	56.0	46.0	43.0	44.0	41.0	23.0	22.0	10.0
			Max	91.0	12.0	2.0	32.0	5.5	40.0	5.0	45.0	62.0	53.0	48.0	48.0	43.0	27.0	27.0	10.0
			CV%	3.6	9.1	15.7	8.8	37.7	7.0	18.3	6.2	5.1	7.5	5.8	4.9	2.4	9.0	11.0	0.0
i	*S. austriaca*	2	**Ar. mean**	**84.5**	**11.0**	**2.5**	**34.5**	**4.5**	**42.0**	**4.0**	**44.0**	**59.0**	**45.5**	**46.5**	**40.5**	**36.5**	**28.5**	**28.0**	**9.0**
			SD	0.7	1.4	0.7	3.5	0.7	1.4	0.0	0.0	1.4	2.1	2.1	0.7	0.7	2.1	1.4	0.0
			Min	84.0	10.0	2.0	32.0	4.0	41.0	4.0	44.0	58.0	44.0	45.0	40.0	36.0	27.0	27.0	9.0
			Max	85.0	12.0	3.0	37.0	5.0	43.0	4.0	44.0	60.0	47.0	48.0	41.0	37.0	30.0	29.0	9.0
			CV%	0.8	12.9	28.3	10.2	15.7	3.4	0.0	0.0	2.4	4.7	4.6	1.7	1.9	7.4	5.1	0.0
j	*S. austriaca*	6	**Ar. mean**	**82.6**	**12.4**	**1.0**	**30.3**	**3.0**	**34.4**	**2.5**	**36.0**	**52.5**	**37.8**	**45.2**	**45.7**	**41.7**	**26.3**	**28.9**	**10.7**
			SD	10.7	1.9	0.6	7.1	1.7	7.2	1.2	5.3	6.5	4.9	3.4	2.3	1.9	2.0	2.2	1.6
			Min	70.0	10.0	0.5	21.0	1.4	25.0	0.8	29.0	45.0	32.0	42.0	42.0	38.8	24.0	26.5	9.0
			Max	97.0	15.0	1.9	38.0	5.0	42.0	3.5	42.0	62.0	45.0	50.0	48.0	44.0	29.0	32.0	13.0
			CV%	13.0	15.3	55.5	23.5	57.0	20.8	49.9	14.8	12.4	13.0	7.5	4.9	4.5	7.5	7.6	15.3
k	*S. pekarovae*	4	**Ar. mean**	**89.5**	**11.5**	**3.9**	**38.0**	**7.5**	**47.8**	**7.8**	**50.0**	**80.3**	**44.8**	**61.3**	**55.5**	**50.0**	**39.8**	**33.5**	**10.5**
			SD	1.7	1.3	2.5	3.5	2.5	3.0	3.0	0.0	0.5	4.1	6.3	5.2	5.1	2.6	5.1	0.6
			Min	87.0	10.0	1.0	35.0	4.0	44.0	5.0	50.0	80.0	40.0	55.0	51.0	45.0	36.0	29.0	10.0
			Max	91.0	13.0	7.0	43.0	10.0	51.0	12.0	50.0	81.0	50.0	70.0	63.0	57.0	42.0	40.0	11.0
			CV%	1.9	11.2	65.3	9.1	33.6	6.3	38.5	0.0	0.6	9.2	10.3	9.4	10.2	6.6	15.1	5.5
l	*S. aria* × *S. austriaca*	3	**Ar. mean**	**75.3**	**12.8**	**3.7**	**35.3**	**3.9**	**41.7**	**4.8**	**38.8**	**60.6**	**36.3**	**55.6**	**47.3**	**40.1**	**31.0**	**34.1**	**7.5**
			SD	8.9	1.5	0.6	9.8	0.1	6.7	1.0	5.9	10.1	5.5	8.1	7.5	4.3	7.8	6.0	0.5
			Min	65.0	11.0	3.0	24.0	3.8	34.0	4.2	32.0	50.0	30.0	50.8	42.8	37.2	22.0	30.2	7.2
			Max	80.5	13.7	4.1	41.0	4.0	45.8	6.0	42.5	70.0	40.0	65.0	56.0	45.0	36.0	41.0	8.0
			CV%	11.8	12.0	16.4	27.7	2.9	16.1	20.9	15.3	16.6	15.1	14.6	15.9	10.7	25.2	17.7	6.2
